# Integrating Rare-Variant Testing, Function Prediction, and Gene Network in Composite Resequencing-Based Genome-Wide Association Studies (CR-GWAS)

**DOI:** 10.1534/g3.111.000364

**Published:** 2011-08-01

**Authors:** Chengsong Zhu, Xianran Li, Jianming Yu

**Affiliations:** Department of Agronomy, Kansas State University, Manhattan, Kansas 66506

**Keywords:** complex trait dissection, association mapping, rare allele, mixed model

## Abstract

High-density array-based genome-wide association studies (GWAS) are complemented by exome sequencing and whole-genome resequencing-based association studies. Here we present a composite resequencing-based genome-wide association study (CR-GWAS) strategy that systematically exploits collective biological information and analytical tools for a robust analysis. We showcased the utility of this strategy by using Arabidopsis (*Arabidopsis thaliana*) resequencing data. Bioinformatic predictions of biological function alteration at each locus were integrated into the process of association testing of both common and rare variants for complex traits with a suite of statistics. Significant signals were then filtered with *a priori* candidate loci generated from genome database and gene network models to obtain *a posteriori* candidate loci. A probabilistic gene network (AraNet) that interrogates network neighborhoods of genes was then used to expand the filtering power to examine the significant testing signals. Using this strategy, we confirmed the known true positives and identified several new promising associations. Promising genes (*AP1*, *FCA*, *FRI*, *FLC*, *FLM*, *SPL5*, *FY*, and *DCL2*) were shown to control for flowering time through either common variants or rare variants within a diverse set of Arabidopsis accessions. Although many of these candidate genes were cloned earlier with mutational studies, identifying their allele variation contribution to overall phenotypic variation among diverse natural accessions is critical. Our rare allele testing established a greater number of connections than previous analyses in which this issue was not addressed. More importantly, our results demonstrated the potential of integrating various biological, statistical, and bioinformatic tools into complex trait dissection.

Genome-wide association studies (GWAS), which have uncovered hundreds of genetic variants associated with complex human diseases and traits, have revolutionized genetic mapping in humans (Altshuler *et al.* 2008; Donnelly 2008; Hindorff *et al.* 2009a) and are being adopted in plants (Atwell *et al.* 2010; Brachi *et al.* 2010). The underlying rationale for GWAS, known as the common disease–common variant (CDCV) hypothesis (Risch and Merikangas 1996), is that common phenotypic variation is caused by common genetic variants. But genes implicated in GWAS often account for only a small fraction of the heritable variation of a phenotype (Hindorff *et al.* 2009b; Manolio *et al.* 2009; Mccarthy *et al.* 2008). Rare functional alleles are among the likely culprits (Pritchard 2001; Reich and Lander 2001) because power to detect association is a function of allele frequency and rare variants are underpowered when sample sizes are limited. In some cases, researchers often exclude single-nucleotide polymorphisms (SNP) that have a minor allele frequency (MAF) less than 5% from association studies (Nordborg *et al.* 2005; Yu *et al.* 2006; Zhao *et al.* 2007). However, recent studies on the frequency of human alleles and their predicted functional effects imply that rare variants (*i.e.*, MAF < 5%) are more likely to be functional than common variants (Gorlov *et al.* 2008), and multiple rare frequency variants together may explain a certain proportion of the genetic variation for certain complex diseases (Bodmer and Bonilla 2008; Johansen *et al.* 2010; Schork *et al.* 2009).

Most of the GWAS in human genetics so far were based on single common variant analyses (Manolio 2010), although it has been shown that multiple rare variants together may account for a few proportions of phenotypic variation for complex diseases (Bansal *et al.* 2010). But these studies with a focus on rare variants were the analysis of one or several candidate genes, and resequenced-based association studies are still not available. Pathway-based approaches have recently been developed to use prior biological knowledge on gene function to facilitate the analysis of GWAS datasets (Wang *et al.* 2010). Up to now, a comprehensive approach that combines statistical analyses of common and rare variants, biological network, function prediction, and other existing methods has not been proposed.

Several notable, critical advances in relevant areas make it feasible to conduct a composite analysis of both common and rare variants beyond the single SNP analysis. First, with next-generation sequencing technologies, exome sequencing or whole-genome resequencing is now possible (Ansorge 2009; Ng *et al.* 2010; Shendure and Ji 2008). Second, biological functions of nucleotide polymorphisms can be predicted with the context sequence of genes (Kumar *et al.* 2009; Ramensky *et al.* 2002) and have been examined in Arabidopsis and rice (Gunther and Schmid 2010). Third, attention has been given to the rare allele issue (Bodmer and Bonilla 2008; Cohen *et al.* 2004; Nejentsev *et al.* 2009), and some specific statistics have been developed to assess the significance of rare variants (Li and Leal 2008; Madsen and Browning 2009; Morgenthaler and Thilly 2007; Morris and Zeggini 2010). Fourth, genome databases and gene networks have been developed to aid the search and confirmation processes of gene-trait associations (Lee *et al.* 2010a; Lee *et al.* 2008; Lee *et al.* 2010b). Comprehensive association analysis calls for an integration of all these advances (Bodmer and Bonilla 2008). In this study, we designed a composite resequencing-based GWAS (CR-GWAS) strategy to integrate these advances, and we showcased the analysis with an Arabidopsis flowering time dataset (Figure 1). We showed specifically how biological function predictions can be incorporated into testing rare variants and broadly how function prediction, genome database, and network information can be integrated into the process of identifying robust associations. With this approach, we identified both common and rare variants underlying variation of flowering time in Arabidopsis.

**Figure 1:**
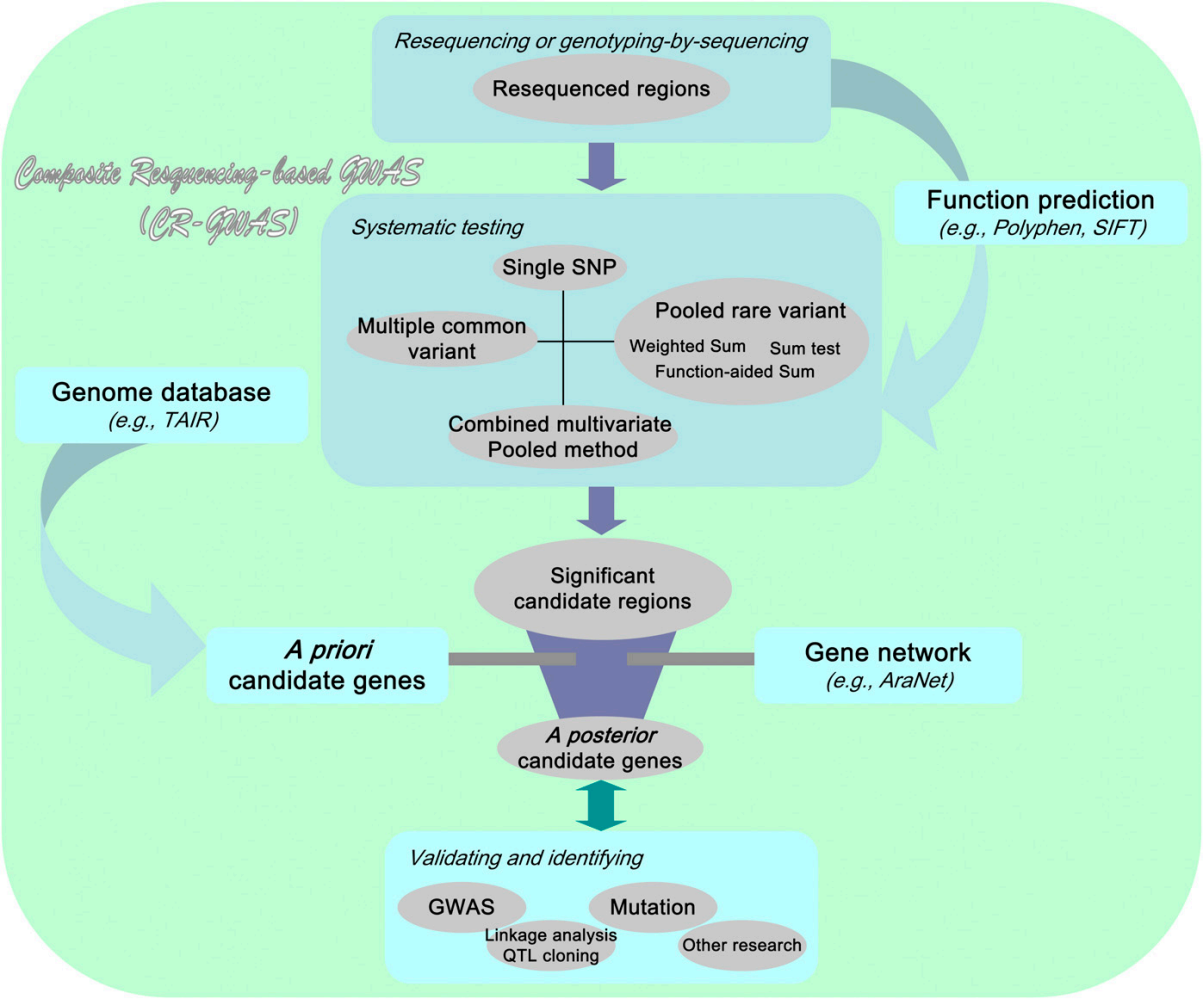
Schematic diagram of a composite resequencing-based GWAS (CR-GWAS) strategy that integrates function prediction, genome database, and gene network information, as well as common variant and rare variant testing.

To date, several GWAS studies with high SNP density have been conducted in plants, including Arabidopsis (Atwell *et al.* 2010; Brachi *et al.* 2010), rice (Huang *et al.* 2010), and maize (Kump *et al.* 2011; Tian *et al.* 2011). These two recent Arabidopsis studies, however, used the array-based genotyping approach, and rare variants (*i.e.*, MAF < 5%) accounted for only 4.7% of all the SNP variants, limiting rare variant analysis. The maize studies involved a genetic design that alters the allele frequency in the final nested association mapping population. The resequencing dataset used in the current study includes data described in earlier publications (Nordborg *et al.* 2005; Zhao *et al.* 2007) and other data of resequenced gene fragments after those publications. To the best of our knowledge, this dataset is the only resequencing-based data with adequate frequencies of rare variants (50%) for a comprehensive analysis in a plant species for which various tools are available. This provides an opportunity to demonstrate the CR-GWAS strategy, particularly the use of rare variant analysis, function prediction, and gene network, which were not conducted in a previous study (Zhao *et al.* 2007). It would be interesting to test this strategy again once data are available from the 1001 Genomes Project.

## Materials and Methods

### Association data

Two resequencing datasets were merged for the current study: one described in earlier publications (Nordborg *et al.* 2005; Zhao *et al.* 2007) and the other based on resequenced gene fragments after initial publications by Magnus Nordborg’s group (Table S4**)**. Additional Arabidopsis requencing data is available at http://walnut.usc.edu/. These 1275 fragments ranged from 454 bp to 942 bp, representing 0.73 Mb of the genome. The average sequence length is 542 bp. All the reliable alignments involving 1116 genes were sequenced across a panel of 96 *Arabidopsis thaliana* lines (Nordborg *et al.* 2005; Zhao *et al.* 2007). A total of 20,810 SNPs were annotated as intergenic, intronic, synonymous, or nonsynonymous to analyze the relationship of allele frequency and function prediction, and then used for association testing. Given the small genome size of Arabidopsis (*i.e.*, about 4% of human genome), the SNP density in this dataset is similar to a study with 500,000-SNP exome or candidate-gene sequence capture in humans. Sixteen traits related to flowering time (Table S1) (Zhao *et al.* 2007) were used for various association testing of common and rare variants. The 3.74% missing entries for phenotypes were imputed using the clustering method (Scheet and Stephens 2006) because of significant correlations among these traits, and the resulting complete data were normalized for association testing.

### Brief description of CR-GWAS

To integrate statistical analyses, function prediction, and gene network, we designed the CR-GWAS strategy (Figure 1). First, we analyzed the gene fragments with a combination of statistical methods to identify significant tests for gene fragments. Second, we examined whether *a priori* candidate genes that were significant at the 0.001 significance level across all methods could be confirmed by previous experiments for genes with common variants. Third, for genes with rare variants (*i.e.*, MAF < 5%), we checked whether the number of functional SNPs on the basis of function prediction within gene fragments was greater than or equal to three. We used three as a cutoff to avoid cases of complete linkage disequilibrium between two SNPs with rare alleles. In addition, we examined the congruency across different statistical methods. Fourth, if the tested genes were not in the *a priori* candidate gene list but were among the top 30 significant tests out of all tests, we searched these genes in the gene network AraNet to verify whether they were connected with *a priori* candidate genes. The detailed procedures are described in the following sections.

### *A priori* candidate genes

A list of 281 *a priori* candidate genes with annotations related to flowering-related traits were retrieved previously from the Arabidopsis Information Resource (TAIR) version 8 (Atwell *et al.* 2010; Brachi *et al.* 2010). Twelve additional genes were retrieved from TAIR 9, resulting in a list of 293 *a priori* candidate genes. Generating the *a priori* candidate gene list is justified because Arabidopsis has been thoroughly studied as a model organism, and its flowering-time pathways have been well characterized. Thirty-five of these *a priori* candidate genes overlapped with genes contained in the 1,275 fragments analyzed for association with flowering time–related traits (Table S1 and Table S4).

### Function prediction

Two approaches based on sequence homology, Polymorphism Phenotype (PolyPhen) (Ramensky *et al.* 2002) and Sorting Intolerant from Tolerant (SIFT) (Kumar *et al.* 2009), were used to evaluate the potential impact of nonsynonymous SNPs. Nonsynonymous SNPs result in amino acid substitutions and are more likely than synonymous SNPs to affect the activity of proteins encoded by the genes. For predictions by PolyPhen, SNPs were classified into three categories: benign, possibly damaging, and probably damaging. Benign SNPs were considered as nonfunctional, whereas possibly or probably protein-damaging SNPs were considered functional. The SNPs predicted to be intolerant by SIFT were considered functional, and SNPs predicted to be tolerant were considered nonfunctional. A nonsynonymous change may be either missense or nonsense. A missense change results in a different amino acid, and a nonsense change results in a premature stop codon. All nonsense SNPs were considered functional because they typically result in more damage to protein structure and function than probably damaging SNPs.

The MAFs were binned into 20 categories in increments of 2.5%, and the various types of SNPs in different MAF bins were tabulated (Table S6**)**. To estimate the relationship between MAF and the proportion of nonsynonymous SNPs predicted to be protein disturbing, power regression ($p^{F}=a\cdot p^{b}$), logarithmic regression ($p^{F}=a\cdot \ln(p)+b$), and linear regression ($p^{F}=a\cdot p+b$) were used to fit the binned data. With predictions from PolyPhen, a power regression function of MAF (*p*), $p^{F}=0.3562{\left(p\right)}^{-1.4162}$, captured 84.6% of the variation of proportion of functional SNPs; this was higher than logarithmic regression (56.9%) or linear regression (27.2%). Similarly, with predictions from SIFT, a power regression function of MAF (*p*), $p^{F}=0.4346{\left(p\right)}^{-1.4863}$, captured 78.3% of the variation, which was higher than logarithmic regression (61.4%) and linear regression (32.2%).

We examined the congruency between function predictions by PolyPhen and SIFT. There was highly significant non-independence (*P-value* = 1.8 × 10^−34^) between the two predictions, driven primarily by the large proportion (66%) of SNPs predicted to be benign by PolyPhen and tolerant by SIFT. Because these two programs were developed using different algorithms, this general congruence observed should be satisfactory.

### Statistical analysis

The unified mixed model was used to control for population structure and relative kinship (Yu *et al.* 2006). The vector of phenotypes, *y*, is modeled as y=Xβ+Zu+e, where β is a vector of subpopulation effects, *i.e.*, Q (STRUCTURE), nonmetric dimensional scaling (nMDS), or principal component analysis (PCA), and *u* is a vector of polygene background effects. *X* contains the coordinates from STRUCTURE, nMDS, and PCA relating *y* to β; *Z* is an incidence matrix of ones and zeros relating *y* to *u*; and *e* is a vector of residual effects. The phenotypic covariance matrix is assumed to have the form $V=2K\sigma_{g}^{2}+I\sigma_{e}^{2}$, where *K* is an *n* × *n* matrix of relative kinship coefficients that define the degree of genetic covariance between a pair of individuals (Loiselle *et al.* 1995), *I* is an *n* × *n* identity matrix, $\sigma_{g}^{2}$ is the genetic variance attributable to genome-wide effects, and $\sigma_{e}^{2}$ is the residual variance. As the effects of population structure on phenotypes varied, we compared the model fit of 22 relevant models across 16 different phenotypes using Bayesian Information Criterion (Yu *et al.* 2006; Zhu and Yu 2009) (Table S2 and Table S3).

With the optimal model for each trait, a GRAMMAR approach was taken in which the adjusted phenotype was computed before testing of common and rare variants to reduce the computational load and avoid convergence issues (Aulchenko *et al.* 2007). For SNPs with MAF greater than 5% (*i.e.*, common variants), a test of association was conducted with adjusted phenotypes by comparing models with and without the specific SNP.

For SNPs with MAF less than 5% (*i.e.*, rare variants), the sum test (Li and Leal 2008; Morris and Zeggini 2010) and weighted sum test (Madsen and Browning 2009) were conducted first. A third test, the function-aided sum test, was adapted by incorporating both biological function prediction (Ramensky *et al.* 2002) and allele frequency into the weighting process (Price *et al.* 2010). In general, the first step for pooling the rare variants is to choose the appropriate genomic units for analysis. One way is to pursue a moving window analysis in which variants in contiguous, possibly overlapping subregions are tested (Bansal *et al.* 2010). In our situation, both collapsed and multivariate tests are confined to the fragment because the 1275 resequenced fragments were mostly independent short segments. We required the number of rare variants with a gene fragment to be greater than or equal to three to be included in the analysis.

For all three tests, the common model was **$y_{i}=\beta_{0}+\beta_{1}z_{i}+e_{i}$**, where *y_i_* is the adjusted phenotype value, β_0_ is the intercept, and β_1_ is the effect of minor allele *vs.* common allele, and *e_i_* is the residual effect. For the sum test, $z_{i}=\sum_{j=1}^{m}\frac{x_{ij}}{m}$, where *m* is the number of rare variants in a gene (or fragment) for *i*th individual, and *x_ij_* denotes the reference allele count of SNP *j* in sample *i*. For the weighted sum test, $z_{i}=\sum_{j=1}^{m}\frac{x_{ij}}{\sqrt{np_{j}(1-p_{j})}}$, where *p_j_* is the frequency of *j*th rare variant and *n* is the population sample size.

For the function-aided sum test, $z_{i}=\sum_{j=1}^{m}S_{j}p_{j}^{F}x_{ij}$, where *S_j_* is independent of allele frequency and is the average probabilistic score of amino acid change from the allele substitution of *j*th rare variant, and *p_j_^F^* is the predicted proportion of functional SNPs with the same MAF frequency of *j*th rare variant. Both *S_j_* and *p_j_^F^* were obtained from the function prediction (Adzhubei *et al.* 2010; Ramensky *et al.* 2002). *S* relates the function class of rare variants to weighting and takes one of three values on the basis of average of delta scores from each category (Table S5): 0.6772 for benign or synonymous, 1.7051 for possibly damaging, and 2.4277 for probably damaging for the Arabidopsis data. The probability score of amino score, *p^F^*, relates allele frequency (*p*) to weighting through the power regression equation described in the previous section, $p^{F}=0.3562{\left(p\right)}^{-1.4162}$. With *S* and *p^F^*, both predicted biological function and allele frequency distribution were introduced into the statistical testing of the rare variants.

For gene fragments with multiple common SNPs, we used the multivariate approach (Pan 2009) in which each variant was assigned the same weight, $z_{i}=\sum_{j=1}^{m}x_{ij}$. For the combined multivariate pooled method, we regarded pooled rare variants (by weighted-sum approach) as individual variants and then applied a multivariate test to analyze groups of variants within a gene fragment.

In the current study, one multiple common variant test and three pooled rare variant tests were examined to determine the significance of the gene fragments and compare the performance of these tests. Likelihood ratio (LR) tests were conducted for all individual methods for single SNP, multiple common variant, sum test, weighted sum test, function-aided sum test, and combined multivariate pooled test. To address multiple testing issues, we used Bonferroni correction to determine significance for the single SNP test because the huge computational load prevented us from using permutation. For all other tests, the experiment-wise LR threshold significance level was determined by computing 1000 permutations (Churchill and Doerge 1994). To compare the results of different tests at the same scale, we calculated the LR/LR99 values.

### Gene network interrogation

With the *a priori* candidate genes (Table S12) as bait, we searched the gene network AraNet (Lee *et al.* 2010a) to find new genes with biological roles inferred by the annotations of the neighbors of these bait genes. AraNet is a probabilistic functional gene network that was constructed for Arabidopsis by a modified Bayesian integration of 24 types of “omics” data from multiple organisms (Lee *et al.* 2010a). The connection between two genes has an associated log-likelihood score that measures the probability of a connection representing a true functional interaction.

In *Arabidopsis thaliana*, flowering time is known to be regulated by a complex genetic network composed of four main converging pathways: the vernalization pathway, the photoperiod pathway, the autonomous pathway, and the gibberellin pathway. These pathways connect physiological and environmental factors, such as photoperiod variation, vernalization, ambient temperature, and plant growth, to promote or repress flowering at an appropriate time (Roux *et al.* 2006). It is known that several genes are involved in these biological networks. After obtaining the list of 293 *a priori* candidate genes, we first checked how many genes are connected by entering these candidate genes as query genes to find their relationships. Then we performed the receiver-operator characteristic (ROC) analysis for the connected genes to further verify their connections. Cross validation (*i.e.*, omitting each seed gene in turn from the seed set) was used, where a higher retrieval rate is given to genes annotated to have the same function cluster in the network (positive) than to genes that are not annotated with that function (negative) in the ROC plot. The degree of the overall connection was summarized by the area under the ROC curve (AUC), ranging from 0.5 to 1 (*i.e.*, genes with high values are deemed to be tightly clustered in a network). In each round, we removed the least-scored gene until the AUC value was greater than or equal to 0.95. If the statistically significant *a priori* candidate genes were in the connected network, they were regarded as the promising candidate genes. In addition, we used these connected *a priori* genes as bait to identify other flowering-time–related genes in AraNet. Then we compared the top 30 significant tests that were not from the list of *a priori* candidate genes with the top 200 (this number was suggested by AraNet) network-connected genes that were retrieved by the bait genes to identify any potentially novel flowering-time–related genes.

## Results

### Distribution of SNPs and function prediction

**Figure 2:**
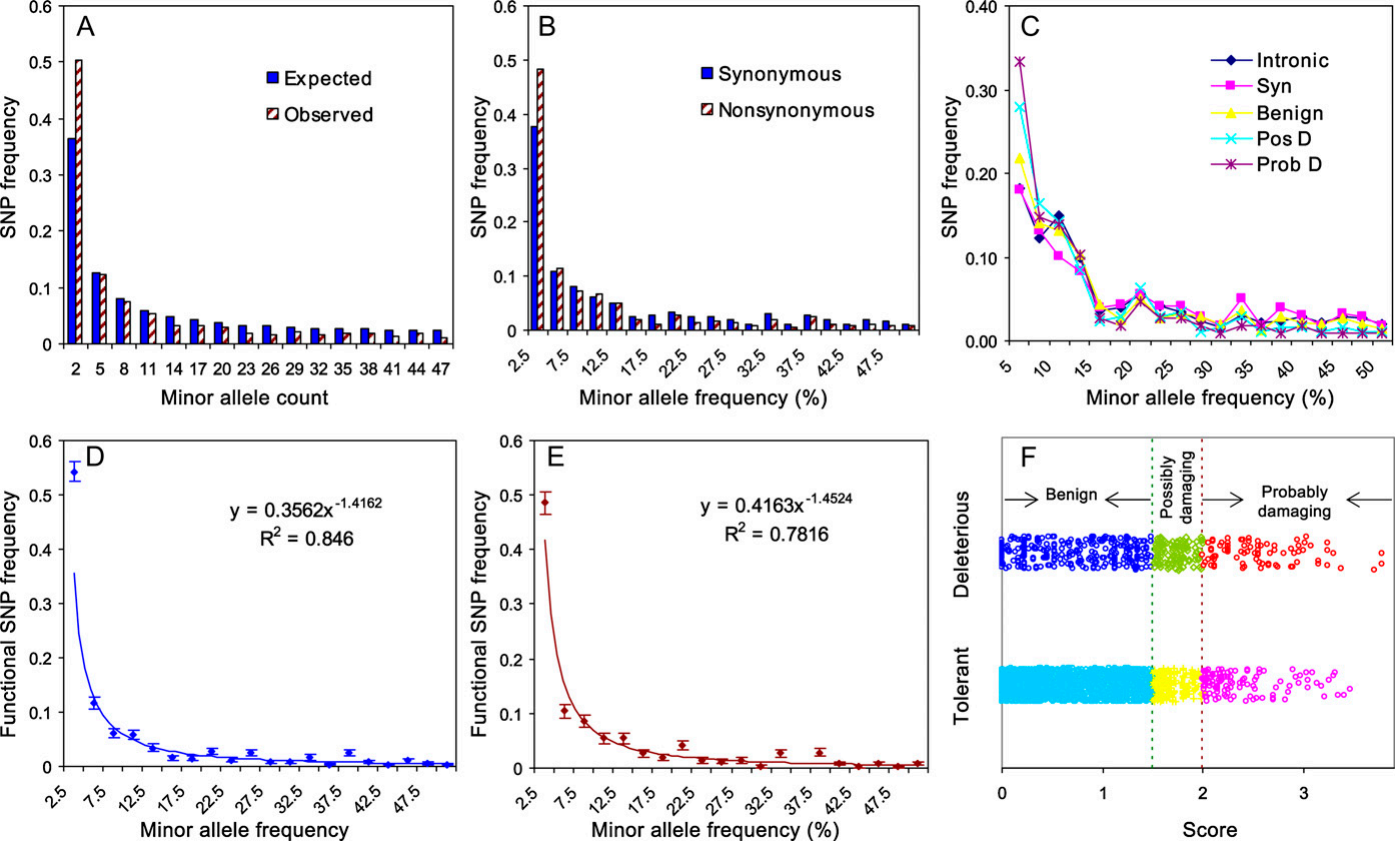
A) SNP frequency in different minor allele count categories; B) distribution of synonymous and nonsynonymous SNPs across different minor allele frequencies; C) distribution of SNPs with different function prediction across different minor allele frequencies; D) PolyPhen-predicted functional SNP frequency across different minor allele frequencies; E) SIFT-predicted functional SNP frequency across different minor allele frequencies; and F) SIFT-predicted function class, deleterious or tolerant, and PolyPhen-predicted score value. SNP, single-nucleotide polymorphism.

To obtain an overall view of the potential function of polymorphisms within gene fragments, we analyzed the SNP frequency distribution and conducted function prediction with PolyPhen (Ramensky *et al.* 2002). First, the distribution of SNPs in different MAF categories showed that the proportion of SNPs with MAF less than 5% (0.5043 ± 0.0026) was significantly higher than the expected value (0.3632 ± 0.0024) under standard population genetics models (Nordborg *et al.* 2005) (Figure 2a). Second, nonsynonymous substitutions were more common than synonymous substitutions for rare SNPs with MAF less than 5% (Figure 2b). In addition, the distributions of SNPs with probably damaging or possibly damaging effects were skewed more to the left than the distributions of SNPs in other categories (Figure 2c, Table S5). The proportion of probably damaging SNPs was highest in the MAF 0–0.05 bin (0.74 ± 0.00045). These results suggest the action of weak purifying selection on amino acids in the *Arabidopsis* *thaliana* genome (Foxe *et al.* 2008; Nordborg *et al.* 2005).

MAF and the proportion of functional SNPs were inversely related in both PolyPhen and SIFT (Kumar *et al.* 2009) predictions (Figure 2d, e). The rapid decrease in the proportion of functional SNPs with increasing MAF was adequately modeled by a power regression function. The congruency (*P-value* = 1.8 × 10^−34^) between the two predictions was mainly driven by the large number of SNPs predicted to be benign by PolyPhen and tolerant by SIFT (Figure 2f). These results demonstrate that a high proportion of SNPs predicted to be functional have low to rare MAF and that analyzing these variants with appropriate statistics would facilitate establishing gene-trait association in GWAS.

### Systematic association testing

We used the mixed model to control for population structure by selecting the optimal model for different traits (Yu *et al.* 2006; Zhu and Yu 2009) (Table S2 and Table S3). Detailed inspection with quantile-quantile plots suggested the need for further adjustment with an inflation factor (Devlin *et al.* 2004). The combination of in- and post-testing adjustments was designed to achieve both accurate individual tests and overall control of false positives (Table S9, Figure S1, Figure S2, Figure S3, Figure S4, Figure S5, Figure S6, Figure S7, Figure S8, Figure S9, Figure S10, Figure S11, Figure S12, Figure S13, Figure S14, Figure S15, and Figure S16). Single SNP tests were conducted for all 20,810 SNPs first. For gene fragments without any SNP with MAF less than 5%, the multiple common variant test was carried out. For gene fragments with SNP with MAF less than 5%, the sum test, weighted sum test, and function-aided sum test were carried out. For gene fragments with a combination of both common and rare variants, a final combined multivariate pooled test was carried out (Table S8).

We examined the predicted function of the significant trait-associated SNPs (TAS). For common variants, 25.6% were nonsynonymous; 10.3%, synonymous; 41.0%, intronic; and 23.1%, intergenic (Table S10). These intronic and intergenic proportions were lower than those in human GWAS results (Hindorff *et al.* 2009b). When adjusted for the base number of each category, 0.042% of the tests for nonsynonymous was significant, which was higher than for synonymous (0.012%), intronic (0.020%), or intergenic (0.033%). Even with the in- and post-testing control, the slightly high number of TASs was not unexpected because that linkage disequilibrium was higher among SNPs with similar allele frequency than among SNPs with different allele frequency (Table S7) and there was a minor allele dependence issue (Table S11) (Brachi *et al.* 2010). In addition, because the LR test has been shown to be liberal (Atwell *et al.* 2010), we conducted additional permutation tests to determine the significance threshold.

### Associations of common variants

Under the assumption of CDCV, we inspected the significant results to identify specific sequence fragments corresponding to genes that were on the list of *a priori* candidate genes for flowering time and that had other biological function evidence. This resulted in four genes with robust associations (Table 1). First, the vernalization-response gene, *FRIGIDA* (*FRI*), has polymorphisms known to affect flowering time through their effect on *FLC* (*FLOWERING LOCUS C*) (Johanson *et al.* 2000; Shindo *et al.* 2005). The *FRI* gene was strongly associated with *FRI* expression levels and was also associated with *FLC* expression levels, consistent with other reports (Atwell *et al.* 2010; Zhao *et al.* 2007). Second, the *FCA* gene, with a function in the posttranscriptional regulation of transcripts involved in the flowering process (Macknight *et al.* 1997), showed significant association with vernalization response to short days [±V(SD)]. The association of *FCA* with flowering time was confirmed in previous analyses (Atwell *et al.* 2010; Brachi *et al.* 2010; Zhao *et al.* 2007). Third, the *FLC* gene, encoding a MADS-domain protein acting as a repressor of flowering time (Ratcliffe *et al.* 2001), showed a significant association with day-length response with vernalization [SD/LD(V)] and short days with 5-week vernalization at University of Southern California (USC) (SDV). Fourth, the floral homeotic gene specifying floral meristem identity in Arabidopsis (Gustafson-Brown *et al.* 1994), *APETALA1* (*AP1*), was associated with long days without vernalization at John Inns Centre (JIC) (JIC0W) and *FLC* expression levels, and it was also associated with long days with 4-week vernalization at John Inns Centre (JIC) (JIC4W) and response to length of vernalization (VERN) if rare variants were considered (Table 1). *AP1* was detected among the 50 best associations in previous GWAS (Brachi *et al.* 2010). Furthermore, *AP1* shares a biological process (GO: 0003700) with *FLC*, and its role in integrating signals from multiple pathways is well established (Mouradov *et al.* 2002). The associations of two additional genes, *CR88* with JIC/USC and *TIC* with JIC4W, need further evidence, although both genes were *a priori* candidate genes involved in the light-dependent pathway (Cao *et al.* 2000) and the circadian clock (Ding *et al.* 2007). A third gene, *DCL2*, containing common variants, is discussed in the association and gene network section.

**Table 1:** Candidate genes with either common or rare variants showing associations to flowering time with the composite resequencing-based association study analysis

Assumption	Gene (Gene ID)	Single SNP Test	Multiple Common	Combined Multivariate Pooled	*A Priori* Candidate Gene	Connected in AraNet	Supporting Evidence
CDCV	*AP1* (AT1G69120)	JIC0W (2.91) FLC (2.85)	JIC0W (3.17) FLC (3.35) JIC4W (3.37) JIC8W (3.09)	JIC0W (3.28) FLC (3.72) JIC4W (3.48) VERN (3.77)	Yes	Yes	Gustafson-Brown *et al.* (1994) [Brachi *et al.* (2010)] Mouradov *et al.* 2002
	*CR88* (AT2G04030)	JIC/USC (1.75)	JIC/USC (2.59)	JIC/USC (2.72)	Yes	No	Cao *et al.* 2000
	*TIC* (AT3G22380)	JIC4W (5.31)	JIC4W (1.87)	JIC4W (3.27)	Yes	No	Ding *et al.* 2007
	*DCL2* (AT3G03300)	SDV (3.08)	SDV (3.55)	SDV (3.94)	No	Yes	Henderson *et al.* 2006
	*FCA* (AT4G16280)	±V(SD) (4.19)	±V(SD) (3.34)	±V(SD) (3.62)	Yes	Yes	Macknight *et al.* 1997 [Atwell *et al.* (2010)] [Brachi *et al.* (2010)] [Zhao *et al.* (2007)]
	*FRI* (AT4G00650)	FRI (14.78) FLC (4.13)	FRI (12.34) FLC (4.77)	FRI (9.43) FLC (3.68) JIC4W (4.23)	Yes	Yes	Johanson *et al.* 2000 Shindo *et al.* 2005 [Atwell *et al.* (2010)] [Zhao *et al.* (2007)]
	*FLC* (AT5G10140)	SD/LD(V) (3.81)	SD/LD(V) (3.14) SDV (3.59)	SD/LD(V) (4.34) SDV (4.81)	Yes	Yes	Ratcliffe *et al.* 2001 [Atwell *et al.* (2010)] [Zhao *et al.* (2007)]
Assumption	Gene (Gene ID)	Sum Test	Weighted Sum	Function-Aided Sum	*A Priori* Candidate Gene	Connected in AraNet	Supporting Evidence
CDRV	*FLM* (AT1G77080)	LD (3.32) JIC2W (4.78) JIC4W (3.23)	JIC2W (1.45)	JIC2W (3.12)	Yes	Yes	Scortecci *et al.* 2001 Werner *et al.* 2005
	*BAS1* (AT2G26710)	LD (4.19) SD (2.91) JIC2W (3.52)	LD (4.55) SD (3.47) JIC2W (3.69)	LD (4.33) SD (3.12) JIC2W (2.23)	Yes	Yes	Turk *et al.* 2005
	*SPL5* (AT3G15270)	JIC/USC (3.22)	JIC/USC (3.47)	JIC/USC (3.57)	Yes	Yes	Wu *et al.* 2009 Wu and Poethig 2006
	*FY* (AT5G13480)	JIC2W (3.55) JIC8W (2.24)	JIC2W (4.05) JIC8W (2.31)	JIC2W (3.67)	Yes	Yes	Simpson *et al.* 2003 [Brachi *et al.* (2010)]

Numbers in parentheses indicate the permutation-derived-log_10_ (*P*-value). References in brackets are genome-wide association studies. CDCV, common disease–common variant; CDRV, common disease–rare variant; SNP, single-nucleotide polymorphism.

If the statistically significant genes were not on the list of *a priori* candidate genes, we considered the top 30 significant tests out of 18,448 tests (16 traits × 1153 fragments) (Table S5, Table S8, and Table S13). Then we checked whether there was at least one significant functional SNP by functional prediction within each fragment. T23J18.17 (AT1G11510) and *SMD1* (AT4G11130) met the requirements. Both genes were associated with SDV (Table S13).

### Associations of rare variants

**Figure 3:**
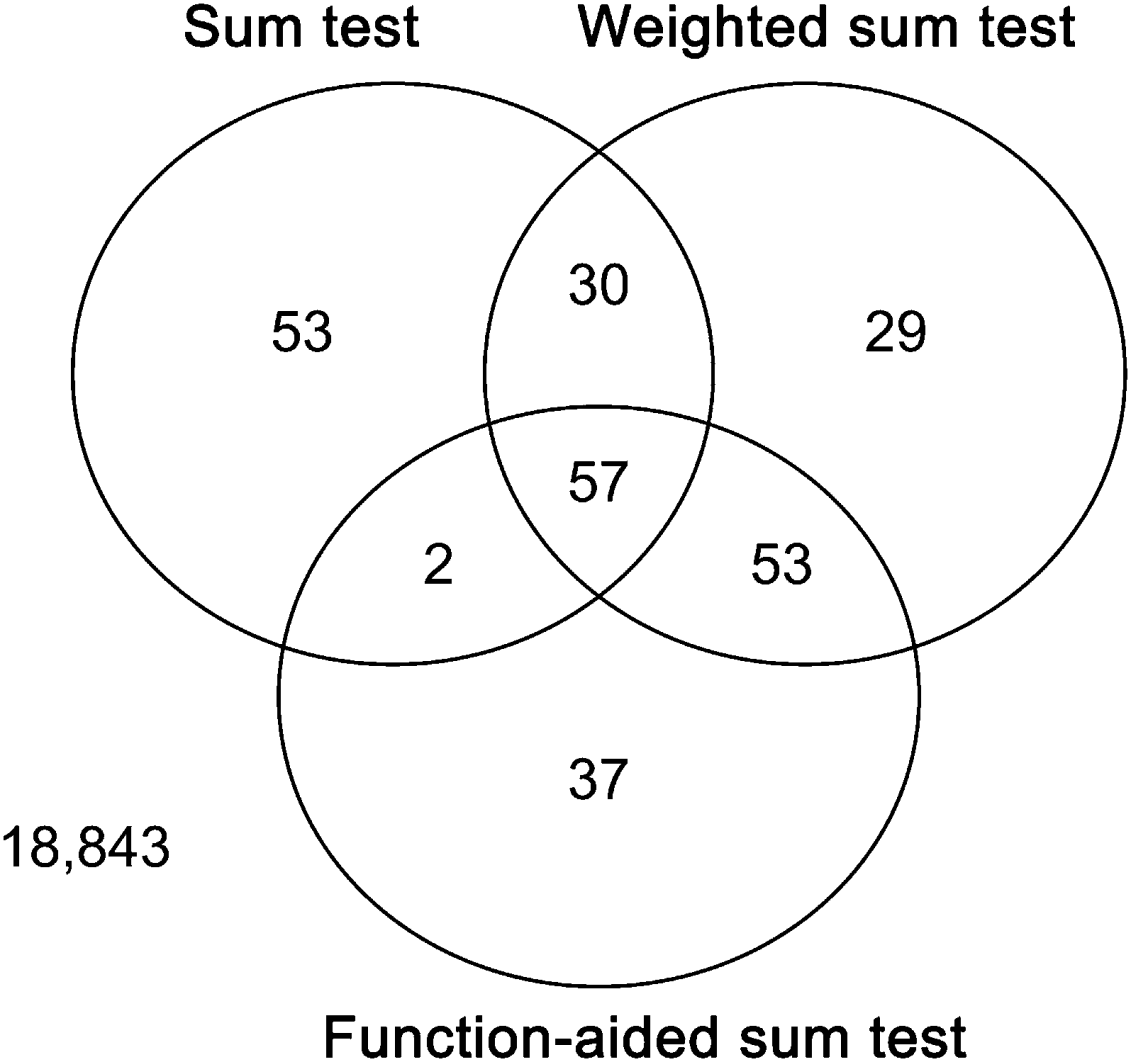
Venn diagram for the number of significant tests from different methods. The numbers in the joined areas indicate the overlap between two or among three methods. The number (18,843) outside of these circles represents tests that are not statistically significant.

**Figure 4:**
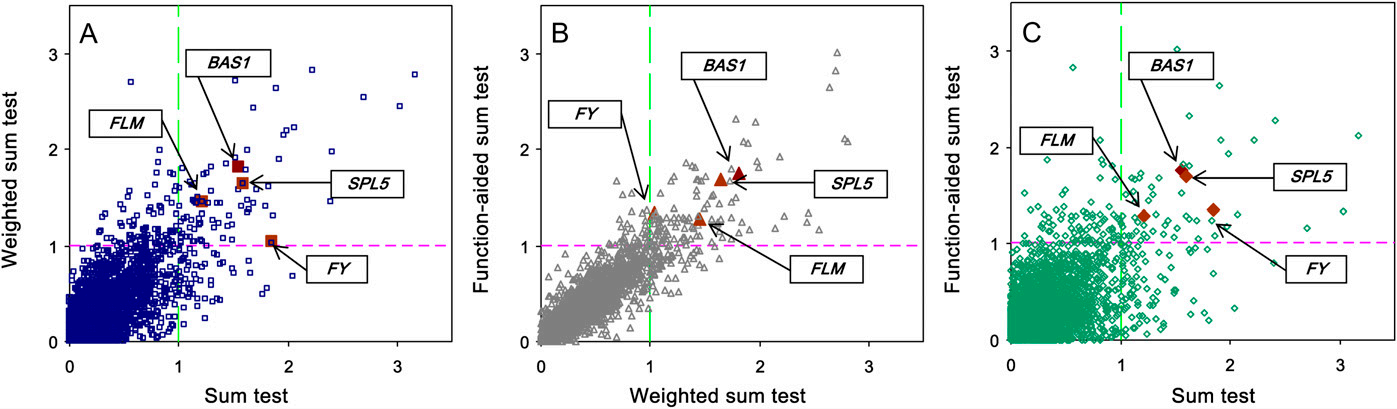
Candidate genes are overrepresented among statistically significant associations. A) LR/LR99 values from the weighted sum test *vs.* sum test; B) LR/LR99 values from the function-aided sum test *vs.* weighted sum test 14; and C) LR/LR99 values from the function-aided sum test *vs.* sum test. Four genes with rare variants (*FLM*, *BAS1*, *SPL5*, and *FY*) are highlighted.

When rare variants were considered, all collapsed methods suggested an excess of significant genes associated with flowering-time–related traits (Figure 4). Unlike the sum test, the weighted sum test and function-aided sum test assign different weights for different MAF. Accordingly, the results for these two methods were more consistent than those for the sum test. Consistence among three methods narrowed down the list of the significant candidate genes, which facilitated the follow-up validation studies (Figure 3). Considering the consistency across pooled rare association methods, 4 of the 35 *a priori* candidate genes showed a significant association with flowering-time–related traits (Table 1, Figure 4, and Table S15). First, *FLOWERING LOCUS M* (*FLM*), a MADS-domain gene that acts as an inhibitor of flowering in Arabidopsis (Scortecci *et al.* 2001), had significant association between pooled rare variants and long days without vernalization at USC (LD) across all methods. However, there was no significant association between common variants within the *FLM* gene and flowering-time–related traits, and *FLM* was not detected in a GWAS with field experiments (Brachi *et al.* 2010). One explanation is that *FLM* contains accession-specific mutations (Werner *et al.* 2005) and is less likely to be detected by the regular methods. Under close examination, we found that rare mutations occur in diverse accessions; this suggests that multiple rare alleles in the *FLM* gene incrementally increase the proportion of genetic variation contributing to flowering time. Second, pooled rare variants in *FY* gene (Simpson *et al.* 2003) were significantly associated with long days with 2-week vernalization at JIC (JIC2W), 4-week vernalization (JIC4W), and 8-week vernalization (JIC8W) (Table 1), agreeing with a previous GWAS (Brachi *et al.* 2010). The difference is that common variants in the *FY* gene were significant in the previous GWAS, but pooled rare variants in the *FY* gene were significant in our study. We suggest that rare genetic variants generate synthetic associations that may have been credited to common variants (Dickson *et al.* 2010). Third, *SPL5* showed significant association with chamber response with vernalization (JIC/USC). *SPL5* and two closely related transcription factors (*SPL3* and *SPL4*) have target sites for MicroRNA *miR156*, and these three genes have overlapping functions in regulating vegetative phase change and floral induction in Arabidopsis (Wu *et al.* 2009; Wu and Poethig 2006). Rare alleles were not addressed in the previous studies, so although *SPL5* was not confirmed in two GWAS reports, it is still a good candidate. Finally, the pooled rare variant in *BAS1* was associated with LD, JIC2W, and short days without vernalization at USC (SD), agreeing with its documented function in photomorphogenesis, hypocotyl elongation, and flowering time (Turk *et al.* 2005). However, caution is warranted because all three SNPs tested were intronic (Table S15).

For gene fragments with rare variants, we identified those fragments that not only contained at least three significant functional SNPs from functional prediction but also were among the top 30 tests (out of 19,104 tests = 16 traits × 1194 fragments) (Table S5 and Table S14). This yielded 4 gene fragments: T9E8.100 (AT4G13360), MXF12.90 (AT5G39080), MWF20.13 (AT5G43420), and K24M7.26 (AT5G52500). All these genes were associated with either JIC/USC or JIC2W. Results from the function-aided sum tests of these genes were also significant.

### Associations and gene network

**Figure 5:**
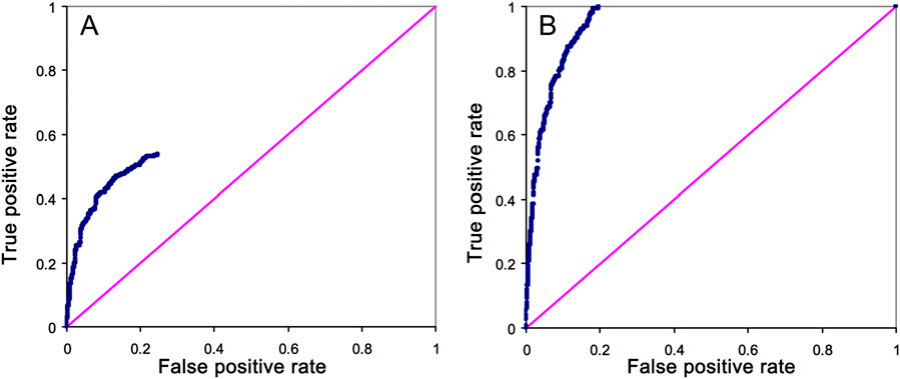
Predictive power of AraNet for flowering-time–related pathways measured by cross-validated receiver-operator characteristics (ROC) curve analyses. A) All 293 *a priori* candidate genes and B) the 150 connected *a priori* candidate genes.

First, we entered 293 *a priori* candidate genes as query genes to find their relationships. The report showed that 161 genes are connected to each other (Table S16), 99 genes disconnected (Table S17), and 33 genes not found in AraNet (Table S18). Verification of these connections by ROC analysis suggested that 150 of these genes should be retained as query genes to identify other flowering-time–related genes within AraNet (Figure 5) because the degree of the overall connection measured by the ROC AUC increased from 0.1013 (293) to 0.9505 (150). Comparing the statistically significant *a priori* genes with these 150 connected genes, we found that *TIC* and *CR88* were not in the network. We then use the 150 connected genes as query genes to identify other flowering-time–related genes. A total of 5501 associated genes, 18 times the original list, were identified and sorted by their log-likelihood scores.

**Figure 6:**
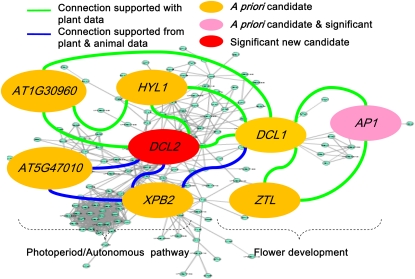
*DCL2* is organized into a network by connecting to *a priori* flowering-time–related genes, evidence for the connections coming from both plant- and animal-derived data sets. Only part of the network is shown. The background is the network constructed with the 150 connected *a priori* candidate genes.

This final list of genes provided additional biological filtering capacity to inspect the statistically significant tests (Table S13, Table S14, and Table S19). Eight of these genes (Table S20 and Table S22) were also among the top 30 statistically significant associations (Table S13 and Table S14). Notably, *DCL2* (AT3G03300, ranked 148th within the 5501 gene list) was significantly associated with SDV (Table 1 and Figure 6).

To determine how *DCL2* is associated with flowering time, we examined its function connections with other *a priori* candidate genes (Figure 6, Table S21 and Table S22). In Figure 6, the left five genes formed a network belonging to the photoperiod/autonomous pathway, and the right three genes formed a flower development biological process (GO accession number: 0009908) (He *et al.* 2010). The two biological processes are linked by *DCL1*. Supporting evidence for the network prediction came primarily from AT-DC (co-occurrence of domains among Arabidopsis proteins) and AT-GN (gene neighborhoods of bacterial and archaeal orthologs of Arabidopsis) with supplementary evidence from HS-DC (co-occurrence of domains among human proteins). Indeed, recent research (not in TAIR7 on which AraNet was built) has shown that *DCL2*, *DCL3*, and *DCL4* redundantly function in RNA-directed DNA methylation and that triple mutants had delayed flowering (Henderson *et al.* 2006).

In summary, 10 candidate genes out of a list of 35 *a priori* candidate genes were determined to have modest to robust associations. Among them, 8 were overlapped by AraNet either through common variant tests (*AP1*, *FCA*, *FRI*, and *FLC*) or through rare variant tests (*FLM*, *FY*, *BAS1*, and *SPL5*). Six other genes (two through common variant tests and 4 through rare variant tests) were determined to be interesting for follow-up studies because they all had top 30 significant tests and supporting evidence from function prediction. Finally, *DCL2* and 7 additional genes had gene network support and statistical significance support.

## Discussion

While some components of this CR-GWAS strategy have been proposed individually, our aim was to bridge advances in different areas. In GWAS, common variants are typically identified though individual testing, whereas rare variants, each with incommensurable effects on phenotypic traits, are difficult to identify using the traditional methods. For multiple rare mutations expected to affect phenotypic traits of interest, grouping variants from the same genes, pathways, and segmental conserved regions has provided promising results (Bodmer and Bonilla 2008; Cohen *et al.* 2004; Nejentsev *et al.* 2009). If various rare variants in a group influence phenotype of complex traits, focusing on the group rather than on an individual variant helps enrich the association signals, reduce the number of degrees of freedom in tests, and subsequently increase statistical power (Mccarthy *et al.* 2008). In the current study, we further factored function prediction and allele frequency distribution into a function-aided sum test of rare variants, establishing a bridge between two research areas: rare allele testing (Li and Leal 2008; Madsen and Browning 2009; Morris and Zeggini 2010) and function prediction (Kumar *et al.* 2009; Ramensky *et al.* 2002). Moreover, we addressed the connection of statistical significance of associate analysis and biological significance via *a priori* candidate genes and a gene network, the combination of which has not been widely explored. Similar gene networks have been constructed for *C. elegans* (WormNet), *S*. *cerevisiae* (YeastNet), *M. musculus* (MouseNet), and *O. sativa* (RiceNet). Individual components (*i.e.*, function prediction, statistical testing for common and rare variants, functional annotation of genomes, and gene network construction) of the composite analysis demonstrated in this study should certainly improve over time, and the overall structure of CR-GWAS should also evolve to accommodate additional components. The ultimate goal is to maximize our capacity in complex trait dissection.

Genetic architecture of flowering time has been extensively studied in the model species Arabidopsis and other plants. The complexity and redundancy involved in controlling the transition from vegetative to reproductive phase involves multiple pathways with many genes (Izawa *et al.* 2003; Komeda 2004). Recent association studies tested whether natural allelic variation of these known genes could account for the flowering-time differences within a diverse collection or derived populations (Atwell *et al.* 2010; Brachi *et al.* 2010; Zhao *et al.* 2007). The allele frequency of genes in the association panel directly affects the signal strength and detection power of standard tests, but this has not been adequately addressed. Following the CR-GWAS strategy, we found that both common and rare variants in a series of genes (*FRI*, *FLC*, *FCA*, *AP1*, *FLM*, *FY*, *SPL5*, and *DCL2*) contribute to the flowering-time variation observed in a diverse collection of Arabidopsis ecotypes. Some additional genes identified through this composite analysis are likely to be further validated.

Although the focus of the current study is on one specific experiment, the proposed approach can be applied quite generally. In the current study, we used resequenced candidate gene fragment data to showcase the CR-GWAS analysis. With the next-generation sequencing technology, we expect similar analysis strategies to be applied to exome sequencing and whole-genome resequencing studies. On the other hand, data generated through array-based genotyping approaches could also be analyzed in a similar framework if the ultrahigh-density genotyping chip containing rare SNPs provides adequate context sequence polymorphisms for function prediction. The capacity of genome databases and gene networks is expected to grow as similar bioinformatics frameworks spread to more species. In addition, incorporating various analytical methods developed for population stratification correction, testing of common variants and rare variants (with flexible weight assignment), threshold determination, and computational load reduction (Aulchenko *et al.* 2007; Devlin *et al.* 2004; Kang *et al.* 2010; Price *et al.* 2006; Pritchard *et al.* 2000; Yu *et al.* 2006; Zhang *et al.* 2010) into a common platform would be challenging but highly desirable.

## Supplementary Material

Supporting Information
