# Supplementary material for: Integrating Rare-Variant Testing, Function Prediction, and Gene Network in Composite Resequencing-Based Genome-Wide Association Studies (CR-GWAS)
Source: G3 (Bethesda). 2011 Aug 1;1(3):233–43. doi: 10.1534/g3.111.000364 (PMC3276137; doi:10.1534/g3.111.000364)
Supplement: Supporting Information [file supp_1_3_233__index.html]

Supporting Information 

# Integrating Rare-Variant Testing, Function Prediction, and Gene Network in Composite Resequencing-Based Genome-Wide Association Studies (CR-GWAS)

## Supporting Information for Zhu *et al.*, 2011

**Files in this Data Supplement:**

- Supporting Information - Figures S1-S16 and Tables S1-S22 (PDF, 1.5 MB)
- Figure S1 - Association analysis results for long days without vernalization at USC (LD) (PDF, 72 KB)
- Figure S2 - Association analysis results for long days with 5-week vernalization at USC (LDV) (PDF, 68 KB)
- Figure S3 - Association analysis results for short days without vernalization at USC (SD) (PDF, 68 KB)
- Figure S4 - Association analysis results for short days with 5-week vernalization at USC (SDV) (PDF, 68 KB)
- Figure S5 - Association analysis results for long days without vernalization at JIC (JIC0W) (PDF, 68 KB)
- Figure S6 - Association analysis results for long days with 2-week vernalization at JIC (JIC2W) (PDF, 72 KB)
- Figure S7 - Association analysis results for long days with 6-week vernalization at JIC (JIC4W) (PDF, 72 KB)
- Figure S8 - Association analysis results for long days with 8-week vernalization at JIC (JIC8W) (PDF, 72 KB)
- Figure S9 - Association analysis results for FLC expression levels (FLC) (PDF, 68 KB)
- Figure S10 - Association analysis results for FRI expression levels (FRI) (PDF, 68 KB)
- Figure S11 - Association analysis results for vernalization response to long days (ratio LD/LDV) (PDF, 68 KB)
- Figure S12 - Association analysis results for vernalization response to short days (ratio SD/SDV) (PDF, 72 KB)
- Figure S13 - Association analysis results for day-length response with vernalization (ratio SDV/LDV) (PDF, 68 KB)
- Figure S14 - Association analysis results for chamber response with vernalization (ratio JIC0W/LD) (PDF, 72 KB)
- Figure S15 - Association analysis results for chamber response without vernalization (ratio JIC4W/LDV) (PDF, 72 KB)
- Figure S16 - Association analysis results for response to length of vernalization (estimated from JIC 0 -8 week data) (PDF, 72 KB)
- Table S1 - Description of phenotypes analyzed in the association tests (PDF, 32 KB)
- Table S2 - Summary of different models used to account for genetic relationship (PDF, 32 KB)
- Table S3 - Model comparisons for 16 Arabidopsis flowering-time related quantitative traits (PDF, 92 KB)
- Table S6 - Counts of SNPs in different MAF categories in the Arabidopsis dataset (PDF, 36 KB)
- Table S7 - Values of *r2* among different bins of allele frequency along various chromosomes (standard deviations in parentheses) (PDF, 44 KB)
- Table S8 - Counts of fragments and SNPs across different chromosomes (PDF, 40 KB)
- Table S9 - Inflation factors by Genomic Control calculated for different statistical methods for 16 flowering time related traits (PDF, 36 KB)
- Table S10 - Number of significant SNPs (MAF>0.5) on the basis of Bonferroni correction there was complete linkage disequilibrium among three rare variants (PDF, 36 KB)
- Table S11 - Number of significant SNPs (MAF<0.5) at a Nominal of 10-5 level (PDF, 36 KB)
- Table S12 - List of the 293 a priori candidate genes for flowering time (PDF, 128 KB)
- Table S13 - Top 30 significant associations between multiple-common variants and flowering-time traits (PDF, 44 KB)
- Table S14 - Top 30 significant associations between pooled-rare variant and flowering-time traits (PDF, 44 KB)
- Table S15 - Rare sequence variations in FLM, SPL5, and FY (PDF, 40 KB)
- Table S16 - Total 161 seed genes are connected one another (PDF, 32 KB)
- Table S17 - Total 99 seed genes are disconnected one another (PDF, 32 KB)
- Table S18 - Total 33 valid Arabidopsis seed genes but NOT found in AraNet (PDF, 28 KB)
- Table S20 - Associated genes to 150 valid seed gene(s) by AraNet that are also among Top 30 statistically significant (PDF, 44 KB)
- Table S21 - 6 Seed genes connected to one another in AraNet (ranked by total connectivity) (PDF, 48 KB)
- Table S22 - Evidence codes for 24 types of data sets incorporated in AraNet (PDF, 36 KB)
- Table S4 - Two candidate resequencing datasets used in the analysis (Microsoft Excel, .xls, 80 KB)
- Table S5 - Function prediction results by PolyPhen and SIFT (Microsoft Excel, .xls, 732 KB)
- Table S19 - 5501 associated genes to 150 valid seed gene(s) by AraNet associated with flowering time (Microsoft Excel, 1.3 MB)
