# Supplementary material for: Integrating Rare-Variant Testing, Function Prediction, and Gene Network in Composite Resequencing-Based Genome-Wide Association Studies (CR-GWAS)
Source: G3 (Bethesda). 2011 Aug 1;1(3):233–43. doi: 10.1534/g3.111.000364 (PMC3276137; doi:10.1534/g3.111.000364)
Supplement: Supporting Information [file supp_1.3.233_FigureS6.pdf]

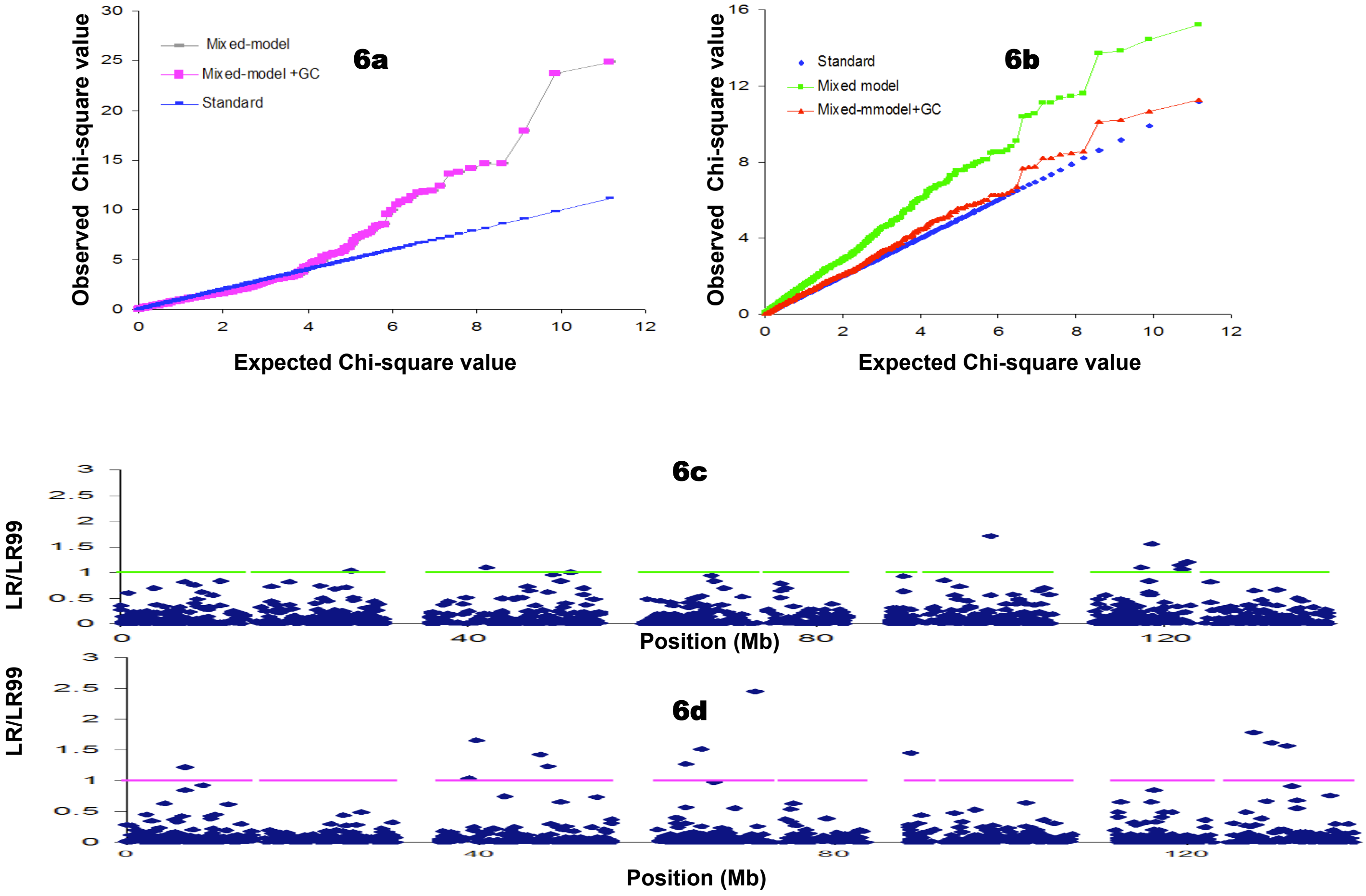

Figure S6 Association analysis results for long days with 2-week vernalization at JIC (JIC2W).

6a. Quantile-quantile plots of chi-square values for multi-SNP simultaneous analysis method;

6b. Quantile-quantile plots of chi-square values for weighted-sum method;

6c. Manhattan plot for multi-SNP simultaneous test along the genome;

6d. Manhattan plot for weighted-sum test along the genome.
