# Supplementary material for: Integrating Rare-Variant Testing, Function Prediction, and Gene Network in Composite Resequencing-Based Genome-Wide Association Studies (CR-GWAS)
Source: G3 (Bethesda). 2011 Aug 1;1(3):233–43. doi: 10.1534/g3.111.000364 (PMC3276137; doi:10.1534/g3.111.000364)
Supplement: Supporting Information [file supp_1.3.233_TableS12.pdf]

Table S12 List of the 293 *a priori* candidate genes for flowering time

Name	LocusTag	TAIR8		TAIR 9		Name	LocusTag	TAIR8		TAIR 9	
		Start	End	Start	End			Start	End	Start	End
CRY2	AT1G04400	1185549	1188516	1185550	1188517	PAP3	AT1G09530	3076584	3079541	3076582	3079539
UFO	AT1G30950	11036161	11037489	11036180	11037508	PHYA	AT1G09570	3095258	3100359	3095256	3100357
AT1G55080	AT1G55080	20556678	20557921	20553011	20554254	HYL1	AT1G09700	3137769	3140355	3137767	3140353
LDL1	AT1G62830	23268155	23270867	23264490	23267202	AT1G10588	AT1G10588	3501146	3501905	3501145	3501904
AP1	AT1G69120	25985993	25989976	25982330	25986313	LWD1	AT1G12190	4394895	4396289	4132967	4134094
FLM	AT1G77080	28960531	28964990	28955637	28960096	DDF1	AT1G12610	4289942	4291015	4289944	4291017
GA2	AT1G79460	29895285	29899480	29890392	29894587	RAV1	AT1G13260	4542165	4543739	4542168	4543742
CAND1	AT2G02560	689787	697595	689788	697596	GAI	AT1G14920	5149221	5151349	5149226	5151354
CR88	AT2G04030	1281838	1286101	1281841	1286104	GA4	AT1G15550	5344473	5346161	5344478	5346166
FVE	AT2G19520	8463018	8466607	8455936	8459525	ATARP4	AT1G18450	6348100	6351968	6348107	6351975
CLF	AT2G23380	9962650	9967439	9955553	9960359	AT1G22690	AT1G22690	8027287	8028114	8027298	8028125
BAS1	AT2G26710	11387570	11390690	11380492	11383612	GI	AT1G22770	8061833	8067705	8061844	8067716
FPA	AT2G43410	18032324	18038320	18025247	18031243	SEPALLATA3	AT1G24260	8593631	8596087	8593642	8596098
CCA1	AT2G46830	19252741	19255983	19245672	19248914	PFT1	AT1G25540	8969052	8974647	8969065	8974660
SPL5	AT3G15270	5140372	5141355	5140365	5141348	CAL	AT1G26310	9100140	9103590	9100153	9103603
VRN1	AT3G18990	6548875	6551859	6548869	6551853	ATGA2OX2	AT1G30040	10537632	10539815	10537648	10539831
HAF2	AT3G19040	6567163	6575288	6567157	6575282	AT1G30960	AT1G30960	11037593	11040014	11037612	11040033
TIC	AT3G22380	7912912	7919517	7912905	7919510	SUF4	AT1G30970	11040262	11043732	11040281	11043751
TEL1	AT3G26120	9547635	9550423	9546398	9549186	FRL2	AT1G31814	11412589	11414483	11412608	11414502
CRP	AT4G00450	203471	211003	202416	211003	VIP1	AT1G43700	16486671	16488681	16484231	16486241
FRI	AT4G00650	269026	271503	269026	271503	ATGA20OX5	AT1G44090	16763117	16764926	16760677	16762486
LD	AT4G02560	1123490	1128421	1123490	1128421	CH1	AT1G44446	16850799	16853664	16848359	16851224
GA1	AT4G02780	1237767	1244813	1237767	1244813	GCR1	AT1G48270	17831621	17834088	17827953	17830420
FCA	AT4G16280	9206613	9214841	9206597	9214825	RTV1	AT1G49480	18317846	18320313	18314178	18316645
VRN2	AT4G16845	9476162	9479897	9476143	9479878	ATGA2OX7	AT1G50960	18893217	18895387	18889549	18891719
AT4G23340	AT4G23340	12195463	12196803	12195453	12196793	AT1G52800	AT1G52800	19667712	19669030	19664044	19665362
FWA	AT4G25530	13038369	13042452	13038360	13042443	SPA4	AT1G53090	19787020	19790570	19783352	19786902
CYP83B1	AT4G31500	15273477	15275316	15273471	15275310	SPL4	AT1G53160	19810087	19811276	19806419	19807608
YAP169	AT5G07200	2243554	2245340	2243553	2245339	ORTH2	AT1G57820	21417835	21421611	21414170	21417946
FLC	AT5G10140	3173498	3179449	3173497	3179448	ARR3	AT1G59940	22069282	22070638	22065617	22066973
FY	AT5G13480	4326531	4331702	4326528	4331699	ATGA20OX4	AT1G60980	22456238	22457805	22452573	22454140
XPB2	AT5G41360	16561568	16566508	16544340	16549280	VIP5	AT1G61040	22486872	22489634	22483207	22485969
DCL1	AT1G01040	23146	31227	23146	31227	ATSCO1	AT1G62750	23237099	23240112	23233434	23236447
MP	AT1G19850	6886879	6891374	6886879	6891374	DDF2	AT1G63030	23371072	23372075	23367407	23368410
CLV2	AT1G65380	24286826	24289249	24286826	24289249	FT	AT1G65480	24335091	24337597	24331428	24333934
LHY	AT1G01060	33379	37840	33379	37840	RGL1	AT1G66350	24751858	24753706	24748195	24750043
CKL13	AT1G04440	1202254	1205802	1202255	1205803	FKF1	AT1G68050	25512339	25514552	25508676	25510889
STO	AT1G06040	1828412	1829889	1828413	1829890	AT1G69935	AT1G69935	26345464	26346789	26341801	26343126

Table 12 (continued)

		TAIR8		TAIR 9				TAIR8		TAIR 9	
Name	LocusTag	Start	End	Start	End	Name	LocusTag	Start	End	Start	End
MMP	AT1G70170	26427537	26429019	26423874	26425356	HOS1	AT2G39810	16619878	16625135	16612800	16618057
AGL12	AT1G71692	26956307	26958789	26952645	26955127	ELF4	AT2G40080	16741372	16741990	16734294	16734912
AT1G72050	AT1G72050	27118686	27121132	27115024	27117470	SHP2	AT2G42830	17827332	17831090	17820255	17824013
HAP2C	AT1G72830	27409118	27411630	27405456	27407969	PIF4	AT2G43010	17893504	17896127	17886427	17889050
MIF1	AT1G74660	28051237	28051788	28047576	28048127	CKB4	AT2G44680	18433621	18435432	18426546	18428357
AT1G74670	AT1G74670	28056947	28057810	28053286	28054149	AGL6	AT2G45650	18811424	18813596	18804350	18806522
ASHH1	AT1G76710	28794623	28797570	28789729	28792676	SOC1	AT2G45660	18814612	18818121	18807538	18811047
EFS	AT1G77300	29044816	29053704	29039922	29048810	SPA1	AT2G46340	19029246	19034486	19022173	19027413
ATGA2OX1	AT1G78440	29516492	29517944	29511599	29513051	APRR9	AT2G46790	19239718	19242156	19232649	19235087
NUA	AT1G79280	29824069	29837871	29819176	29832978	AT2G47310	AT2G47310	19430754	19434347	19423684	19427277
ELF7	AT1G79730	30005431	30008898	30000538	30004005	RFI2	AT2G47700	19559384	19561654	19552314	19554584
ATGA3OX4	AT1G80330	30202953	30204429	30198061	30199537	MBD9	AT3G01460	173323	182461	173316	182454
GA4H	AT1G80340	30205585	30207092	30200693	30202200	SEPALLATA2	AT3G02310	464286	467081	464279	467074
MOS3	AT1G80680	30328900	30333661	30324008	30328769	COL2	AT3G02380	487243	488700	487236	488693
RGA1	AT2G01570	255248	257549	255249	257550	GASA5	AT3G02885	638028	639062	638021	639055
PKS1	AT2G02950	854946	856537	854947	856538	ATVGT1	AT3G03090	700463	704776	700456	704769
SEPALLATA4	AT2G03710	1129265	1131835	1129268	1131838	AT3G04510	AT3G04510	1215642	1216964	1215636	1216958
ELF8	AT2G06210	2428900	2436684	2428903	2436687	FLK	AT3G04610	1250559	1254879	1250553	1254873
AT2G14900	AT2G14900	6411257	6412412	6404175	6405330	HST	AT3G05040	1401277	1408203	1401271	1408197
PHYB	AT2G18790	8146963	8151512	8139881	8144430	ATGID1A	AT3G05120	1430477	1432784	1430471	1432778
LKP2	AT2G18915	8201654	8204565	8194572	8197483	ATHAP2B	AT3G05690	1676552	1678938	1676546	1678932
PIL5	AT2G20180	8711105	8713973	8704024	8706892	AT3G06910	AT3G06910	2178636	2181203	2178630	2181197
FIO1	AT2G21070	9047944	9050609	9040863	9043528	AtPRMT4b	AT3G06930	2185149	2189393	2185143	2189387
SVP	AT2G22540	9586954	9590973	9579874	9583893	COL9	AT3G07650	2441663	2444538	2441657	2444532
AGL17	AT2G22630	9625452	9629037	9618372	9621957	AT3G10185	AT3G10185	3145584	3146204	3145579	3146199
COL3	AT2G24790	10573977	10575224	10566898	10568145	FLD	AT3G10390	3229298	3231824	3229293	3232345
ELF3	AT2G25930	11066113	11070402	11059035	11063324	ATMYB65	AT3G11440	3602099	3605110	3602093	3605104
ATC	AT2G27550	11780328	11781758	11773251	11774681	SPY	AT3G11540	3631893	3637961	3631887	3637955
SYD	AT2G28290	12063290	12080160	12056213	12073083	PIE1	AT3G12810	4065049	4074085	4065042	4074078
TOE1	AT2G28550	12233028	12235620	12225951	12228543	LDL2	AT3G13682	4479200	4481516	4479193	4481509
AT2G30810	AT2G30810	13134903	13135743	13127826	13128666	SPA3	AT3G15354	5169102	5172844	5169095	5172837
COP1	AT2G32950	13985010	13990612	13977933	13983535	ATFYPP3	AT3G19980	6961742	6965114	6961736	6965108
SPL3	AT2G33810	14312077	14313148	14305001	14306072	DDL	AT3G20550	7174470	7177948	7174464	7177942
FES1	AT2G33835	14318863	14321776	14311787	14314700	FIE	AT3G20740	7248815	7252458	7248809	7252452
ATGA2OX3	AT2G34555	14564067	14565776	14556988	14558697	AT3G21320	AT3G21320	7499059	7501847	7499053	7501841
FHY1	AT2G37678	15808543	15809871	15801465	15802793	VRN5	AT3G24440	8876034	8878178	8876027	8878171
SNZ	AT2G39250	16395964	16398151	16388886	16391073	LWD2	AT3G26640	9794457	9795694	9793220	9794457
AT2G39540	AT2G39540	16507944	16508319	16500866	16501241	FUS3	AT3G26790	9855065	9857226	9853828	9855989

Table 12 (continued)

		TAIR8		TAIR 9				TAIR8		TAIR 9	
Name	LocusTag	Start	End	Start	End	Name	LocusTag	Start	End	Start	End
BR6OX2	AT3G30180	11813216	11816244	11810737	11813765	ATGA2OX8	AT4G21200	11302695	11306611	11302685	11306601
ATARP6	AT3G33520	14104642	14106535	14093656	14095549	ATGA3OX3	AT4G21690	11527241	11529072	11527229	11529060
PCL1	AT3G46640	17194075	17196203	17183090	17185218	EBS	AT4G22140	11727738	11730521	11727726	11730509
CDF3	AT3G47500	17514985	17517043	17504000	17506058	AGL19	AT4G22950	12023926	12027432	12023915	12027421
REF6	AT3G48430	17946594	17951731	17935609	17940746	AGL24	AT4G24540	12670975	12674082	12670965	12674072
AMP1	AT3G54720	20265703	20268826	20254725	20257848	PGI1	AT4G24620	12708762	12712835	12708752	12712825
SMZ	AT3G54990	20384695	20387499	20373718	20376522	GA5	AT4G25420	12990894	12992468	12990884	12992458
AGL16	AT3G57230	21188689	21191911	21177423	21180932	TOR1	AT4G27060	13581407	13585161	13581401	13585155
AT3G57300	AT3G57300	21210467	21218864	21199488	21207885	CIP7	AT4G27430	13718685	13723330	13718679	13723324
AGL18	AT3G57390	21244678	21246888	21233701	21235911	ATHXK1	AT4G29130	14352043	14355109	14352037	14355103
GIS	AT3G58070	21517590	21518631	21506613	21507654	VIP3	AT4G29830	14597667	14599306	14597661	14599300
SHP1	AT3G58780	21749437	21752884	21738460	21741907	AT4G30200	AT4G30200	14786639	14790509	14786633	14790503
PIL6	AT3G59060	21838955	21841484	21827978	21830507	ATPRMT5	AT4G31120	15132017	15136645	15132011	15136639
CKB3	AT3G60250	22281312	22283088	22270337	22272113	FLP1	AT4G31380	15229791	15230724	15229785	15230718
AGL13	AT3G61120	22629234	22631466	22618259	22620491	KNAT5	AT4G32040	15494071	15496362	15494065	15496356
PIL2	AT3G62090	22999522	23001684	22988547	22990709	ATH1	AT4G32980	15914725	15918047	15914722	15918044
ATGID1B	AT3G63010	23300400	23302461	23289425	23291486	AT4G33280	AT4G33280	16047358	16049359	16047354	16049355
AT4G00690	AT4G00690	281645	283129	281645	283129	FD	AT4G35900	17004598	17006290	17004595	17006287
ETC3	AT4G01060	460472	461085	460472	461085	AP2	AT4G36920	17400844	17403329	17400847	17403332
EZA1	AT4G02020	886600	891955	886600	891955	HLS1	AT4G37580	17658606	17660872	17658612	17660878
DFL2	AT4G03400	1497535	1499864	1497536	1499865	AGL21	AT4G37940	17835689	17838615	17835695	17838621
PDF2	AT4G04890	2476487	2482343	2476489	2482345	BRI1	AT4G39400	18324655	18328820	18324661	18328826
CRY1	AT4G08920	5724100	5727250	5724103	5727253	FHL	AT5G02200	437458	438892	437460	438894
GASA2	AT4G09610	6074767	6075642	6074770	6075645	PRR7	AT5G02810	637895	641975	637897	641977
SPA2	AT4G11110	6771601	6777221	6771605	6777225	LCL1	AT5G02840	648702	651970	648704	651972
AGL14	AT4G11880	7143109	7147216	7143115	7147222	ATHB51	AT5G03790	1004982	1006372	1004983	1006373
pEARLI 1	AT4G12480	7406102	7406934	7406105	7406937	TFL1	AT5G03840	1024640	1025811	1024641	1025812
COP9	AT4G14110	8132881	8134915	8132886	8134920	ELF6	AT5G04240	1169545	1174879	1169544	1174878
ELIP2	AT4G14690	8418278	8419258	8418283	8419263	CPD	AT5G05690	1702689	1706788	1702688	1706787
FAR1	AT4G15090	8614063	8618142	8614067	8618145	MYB33	AT5G06100	1837908	1840728	1837907	1840727
AT4G15180	AT4G15180	8651406	8662587	8651406	8662587	CHE	AT5G08330	2680745	2681814	2680744	2681813
ESD4	AT4G15880	9012660	9016131	9012645	9016116	AT5G10625	AT5G10625	3358788	3359782	3358787	3359781
PHYD	AT4G16250	9195617	9199501	9195602	9199486	HY5	AT5G11260	3593381	3594993	3593380	3594992
HAT4	AT4G16780	9449133	9450762	9449114	9450743	EMF1	AT5G11530	3695863	3701549	3695862	3701548
AT4G16810	AT4G16810	9459889	9462272	9459870	9462253	HAP2A	AT5G12840	4050694	4053609	4050691	4053606
CKB2	AT4G17640	9825210	9827285	9825197	9827272	AGL15	AT5G13790	4449017	4450846	4449014	4450843
PHYE	AT4G18130	10042149	10046094	10042137	10046082	AT5G14920	AT5G14920	4826482	4827983	4826479	4827980
TSF	AT4G20370	11000782	11003007	11000771	11002996	GASA4	AT5G15230	4944903	4946219	4944900	4946216

Table 12 (continued)

		TAIR8		TAIR 9				TAIR8		TAIR 9	
Name	LocusTag	Start	End	Start	End	Name	LocusTag	Start	End	Start	End
CO	AT5G15840	5171185	5172761	5171182	5172758	VIP2	AT5G59710	24074633	24079144	24057407	24061918
COL1	AT5G15850	5176094	5177900	5176091	5177897	AT5G59845	AT5G59845	24128550	24129246	24111324	24112020
KIN1	AT5G15960	5209901	5210730	5209898	5210727	APRR3	AT5G60100	24215225	24218590	24197999	24201364
KIN2	AT5G15970	5211914	5212668	5211911	5212665	TOE2	AT5G60120	24225012	24228950	24207786	24211724
FRL1	AT5G16320	5344505	5346022	5344502	5346019	AGL8	AT5G60910	24519708	24523369	24502482	24506143
TFL2	AT5G17690	5827173	5829684	5827171	5829682	VIP4	AT5G61150	24620882	24624951	24603656	24607725
ASP2	AT5G19550	6598019	6601821	6598017	6601819	TOC1	AT5G61380	24692290	24695776	24675064	24678550
NPH4	AT5G20730	7016447	7022115	7016445	7022113	LFY	AT5G61850	24861521	24864159	24844295	24846933
HUA2	AT5G23150	7785838	7792492	7785835	7792489	AT5G62040	AT5G62040	24940036	24940935	24922810	24923709
APRR5	AT5G24470	8355954	8358876	8355951	8358873	CDF1	AT5G62430	25086319	25088160	25069093	25070934
FPF1	AT5G24860	8541781	8542452	8541778	8542449	ELF5	AT5G62640	25166659	25169767	25149433	25152541
TNY	AT5G25810	8986774	8987790	8986771	8987787	LIP1	AT5G64813	25927505	25930122	25910279	25912896
GA3	AT5G25900	9036021	9038409	9036018	9038406	AGL31	AT5G65050	25999480	26003552	25982254	25986326
AT5G27230	AT5G27230	9584095	9588052	9584092	9588049	MAF3	AT5G65060	26004655	26008541	25987429	25991315
ATGID1C	AT5G27320	9629090	9631213	9629087	9631210	MAF4	AT5G65070	26009486	26013360	25992260	25996134
AT5G28450	AT5G28450	10372942	10374194	10372938	10374190	AGL68	AT5G65080	26014730	26019691	25997504	26002465
LSH1	AT5G28490	10454397	10455200	10454393	10455196	AT5G65540	AT5G65540	26212915	26215548	26195689	26198322
PHYC	AT5G35840	14025056	14028994	14007826	14011764	ICU2	AT5G67100	26794220	26802330	26776994	26785104
TCH2	AT5G37770	15016084	15016849	14998854	14999619	UBC1	AT1G14400	4928533	4927011	4928533	4927011
PMI15	AT5G38150	15240346	15242177	15223116	15224947	CIB5	AT1G26260	9087104	9089378	9087104	9089378
CDF2	AT5G39660	15895927	15898272	15878699	15881044	SEU	AT1G43850	16617152	16622049	16617152	16622049
CIP1	AT5G41790	16744758	16750075	16727530	16732847	GRF2	AT1G78300	14095549	14093656	14095549	14093656
CUL4	AT5G46210	18748645	18754037	18731418	18736810	HTA11	AT3G54560	20196266	20197650	20196266	20197650
AT5G46910	AT5G46910	19065007	19068107	19047780	19050880	LUG	AT4G32551	15707510	15713579	15707510	15713579
LBA1	AT5G47010	19089236	19096561	19072009	19079334	SEPALLATA1	AT5G15800	5154154	5151334	5154154	5151334
CKB1	AT5G47080	19141839	19143838	19124612	19126611	SEF	AT5G37055	14642440	14641551	14642440	14641551
PAT1	AT5G48150	19539481	19541924	19522255	19524698	ARF8	AT5G37020	14630028	14634387	14630028	14634387
AtPRMT4a	AT5G49020	19888477	19892146	19871251	19874920						
EMF2	AT5G51230	20840962	20846790	20823736	20829564						
AT5G51310	AT5G51310	20870080	20871944	20852854	20854718						
AT2353	AT5G51810	21072414	21074034	21055188	21056808						
PGM	AT5G51820	21080594	21085283	21063368	21068057						
DFL1	AT5G54510	22148319	22150904	22131093	22133678						
ZTL	AT5G57360	23258653	23261816	23241427	23244590						
VIN3	AT5G57380	23263621	23266730	23246395	23249504						
MSI1	AT5G58230	23573238	23575471	23556012	23558245						
SRR1	AT5G59560	24017782	24019183	24000556	24001957						
AT5G59570	AT5G59570	24021114	24022738	24003888	24005512						
