# Supplementary material for: Integrating Rare-Variant Testing, Function Prediction, and Gene Network in Composite Resequencing-Based Genome-Wide Association Studies (CR-GWAS)
Source: G3 (Bethesda). 2011 Aug 1;1(3):233–43. doi: 10.1534/g3.111.000364 (PMC3276137; doi:10.1534/g3.111.000364)
Supplement: Supporting Information [file supp_1.3.233_FigureS15.pdf]

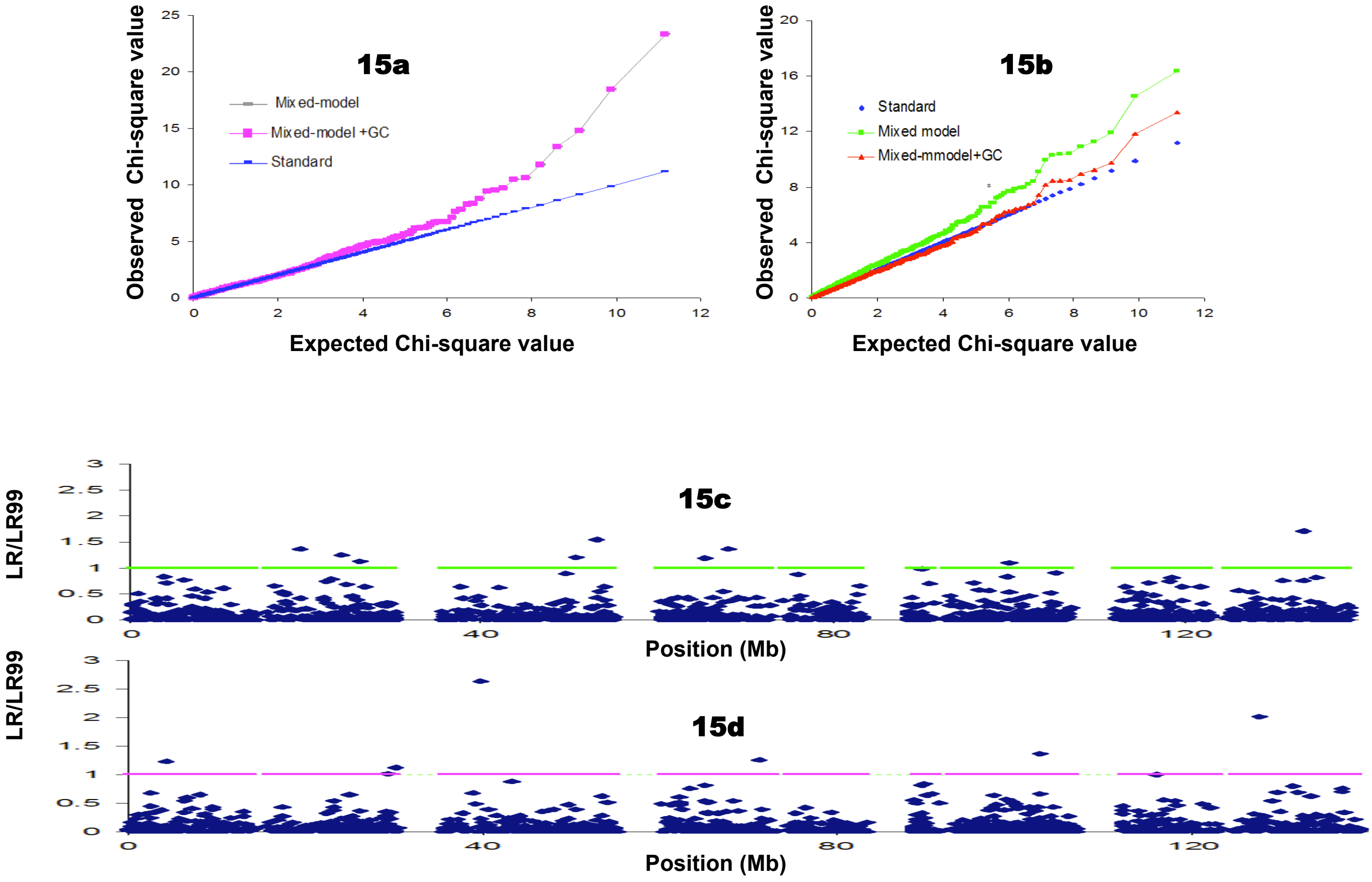

Figure S15 Association analysis results for chamber response without vernalization (ratio JIC4W/LDV).

15a. Quantile-quantile plots of chi-square values for multi-SNP simultaneous analysis method;

15b. Quantile-quantile plots of chi-square values for weighted-sum method;

15c. Manhattan plot for multi-SNP simultaneous test along the genome;

15d. Manhattan plot for weighted-sum test along the genome.
