# Supplementary material for: Integrating Rare-Variant Testing, Function Prediction, and Gene Network in Composite Resequencing-Based Genome-Wide Association Studies (CR-GWAS)
Source: G3 (Bethesda). 2011 Aug 1;1(3):233–43. doi: 10.1534/g3.111.000364 (PMC3276137; doi:10.1534/g3.111.000364)
Supplement: Supporting Information [file supp_1.3.233_TableS1.pdf]

Table S1 Description of phenotypes analyzed in the association tests

Phenotype	Description
	Flowering time
LD	Long days without vernalization at USC
LDV	Long days with 5-week vernalization at USC
SD	Short days without vernalization at USC
SDV	Short days with 5-week vernalization at USC
JIC0W	Long days without vernalization at JIC
JIC2W	Long days with 2-week vernalization at JIC
JIC4W	Long days with 4-week vernalization at JIC
JIC8W	Long days with 8-week vernalization at JIC
Gene expression	
FLC	FLC expression
FRI	FRI expression
Estimated trait	
±V (LD)	Vernalization response to long days (ratio LD/LDV)
±V(SD)	Vernalization response to short days (ratio SD/SDV)
SD/LD(V)	Day-length response with vernalization (ratio SDV/LDV)
JIC/USC	Chamber response with vernalization (ratio JIC0W/LD)
JIC/USC(V)	Chamber response without vernalization (ratio JIC4W/LDV)
VERN	Response to length of vernalization (estimated from JIC 0 -8 week data)
