# Supplementary material for: Integrating Rare-Variant Testing, Function Prediction, and Gene Network in Composite Resequencing-Based Genome-Wide Association Studies (CR-GWAS)
Source: G3 (Bethesda). 2011 Aug 1;1(3):233–43. doi: 10.1534/g3.111.000364 (PMC3276137; doi:10.1534/g3.111.000364)
Supplement: Supporting Information [file supp_1.3.233_TableS2.pdf]

Table S2 Summary of different models used to account for genetic relationship

Model	Description
Simple	Regression model without any correction
Q	Regression model with fixed population structure covariates
PCA	Regression model with fixed principal component covariates
nMDS	Regression model with fixed nonmetric multidimensional scaling covariates
K	Mixed model with random kinship
Q+K	Mixed model with fixed population structure covariates and random kinship
PCA+K	Mixed model with fixed principal component covariates and random kinship
nMDS+K	Mixed model with fixed nonmetric multidimensional scaling covariates and random kinship
