# Supplementary material for: Integrating Rare-Variant Testing, Function Prediction, and Gene Network in Composite Resequencing-Based Genome-Wide Association Studies (CR-GWAS)
Source: G3 (Bethesda). 2011 Aug 1;1(3):233–43. doi: 10.1534/g3.111.000364 (PMC3276137; doi:10.1534/g3.111.000364)
Supplement: Supporting Information [file supp_1.3.233_TableS3.pdf]

Table S3 Model comparisons for 16 Arabidopsis flowering-time related quantitative traits

Models	LD		LDV		SD		SDV	
	Deviance	BIC	Deviance	BIC	Deviance	BIC	Deviance	BIC
Simple	268.6	277.7	273.3	282.4	269.1	278.2	272.1	281.2
K	214.1	227.8	-	-	234.7	248.4	250.9	264.6
Q8	152.8	198.4	183.5	229	166.7	212.3	221.8	267.3
Q8+K	134.7	184.8	-	-	164.5	214.6	217.6	267.7
nMDS8	150	195.5	172.4	217.9	180.6	226.2	191.6	**237.1**
nMDS+K	141.1	191.2	168.8	218.8	179.1	229.2	-	-
PCA1	238.8	252.4	258.1	271.8	265	278.7	266.6	280.3
PCA1+K	193.1	211.3	-	-	232.2	250.4	248.6	266.9
PCA2	174.3	192.6	216.7	234.9	228.8	247	239.9	258.1
PCA2+K	154.5	**177.2**	196	218.8	208.5	231.2	234.1	256.8
PCA3	165	187.8	207.3	230.1	183.8	206.6	232.9	255.6
PCA3+K	-	-	-	-	178.1	**205.4**	227.7	255
PCA4	164.6	191.9	205.6	232.9	182.2	209.6	230.7	258.1
PCA4+K	-	-	-	-	176.5	208.4	224.2	256.1
PCA5	164	195.9	200.7	232.6	181.6	213.5	219.3	251.2
PCA5+K	-	-	-	-	176.2	212.6	216.2	252.6
PCA6	156.6	193	183.6	220	177.2	213.7	215.1	251.5
PCA6+K	-	-	-	-	173	214	213.4	254.4
PCA7	144.1	185	166.6	**207.6**	165	206	208.9	249.9
PCA7+K	134.9	180.5	163.8	209.4	164.3	209.8	208.7	254.3
PCA8	143.2	188.7	166.3	211.9	164.2	209.8	208.5	254.1
PCA8+K	133.2	183.3	163.1	213.2	163.6	213.7	208.4	258.5

Models	JIC0W		JIC2W		JIC4W		JIC8W	
	Deviance	BIC	Deviance	BIC	Deviance	BIC	Deviance	BIC
Simple	276.2	285.3	271.7	280.8	267.4	276.5	276.3	285.4
K	246.1	259.8	232.3	246	231.6	245.3	258.3	272
Q8	187	232.6	155.3	200.8	164.7	210.2	197.5	243.1
Q8+K	180.2	**230.3**	149.4	199.5	159.6	209.7	197.4	247.5
nMDS8	189.5	235.1	154.5	200.1	170.6	216.1	211.1	256.6
nMDS+K	188	238.1	153.8	203.9	168.4	218.5	210.4	260.5
PCA1	264.7	278.4	254.8	268.4	255.3	269	276.3	290
PCA1+K	240.2	258.4	224.5	242.7	226.6	244.9	258.3	276.5
PCA2	224.8	243.1	193.2	211.4	199.7	218	261.1	279.3
PCA2+K	216.3	239.1	184.9	207.7	191.8	214.6	250	272.7
PCA3	204.4	227.1	161.3	184.1	168.5	**191.2**	213.5	236.3
PCA3+K	197.7	225	155	**182.4**	164	191.3	210.6	237.9
PCA4	203.3	230.6	160	187.3	165.4	192.7	205.1	**232.4**
PCA4+K	197.2	229	153.4	185.2	160.5	192.4	204.5	236.4
PCA5	202	233.9	159.8	191.7	165.3	197.2	205	236.8

PCA5+K	194.6	231	153.4	189.8	-	-	204.5	240.9
PCA6	194	230.5	155.5	191.9	163.5	199.9	200.9	237.3
PCA6+K	190.3	231.3	151.2	192.1	159.8	200.7	-	-
PCA7	193.5	234.5	149.3	190.3	158.4	199.4	198.8	239.8
PCA7+K	190.3	235.8	147.2	192.8	156.5	202	-	-
PCA8	191.2	236.8	148.6	194.1	158.4	203.9	198.3	243.9
PCA8+K	186.3	236.4	146.1	196.2	156.2	206.3	-	-

Models	FRI		FLC		±V(LD)		±V(SD)	
	Deviance	BIC	Deviance	BIC	Deviance	BIC	Deviance	BIC
Simple	268.5	277.8	268.8	277.4	269.8	278.9	267	276.1
K	266.9	280.5	249.6	263.2	227.4	241.1	255.7	**269.4**
Q8	242.3	287.8	232.6	278.1	186.8	232.3	246.8	292.4
Q8+K	-	-	228.8	278.8	170.2	220.3	246.6	296.7
nMDS8	238.1	283.7	213.9	259.5	189.1	234.6	240.3	285.9
nMDS+K	-	-	-	-	-	-	-	-
PCA1	260.7	**274.4**	261	274.7	243.4	257	266.7	280.3
PCA1+K	260.6	278.8	246.8	265	209.6	227.8	255.1	273.3
PCA2	259.6	277.8	244	262.2	201.3	219.5	266.7	284.9
PCA2+K	259.6	282.3	236.7	259.4	187.7	**210.5**	-	-
PCA3	258.7	281.5	243	265.8	196.1	218.9	256.2	278.9
PCA3+K	258.6	285.9	236.1	263.4	-	-	255.3	282.6
PCA4	256.7	284	236.3	263.6	196.1	223.4	253.5	280.9
PCA4+K	256.6	288.5	234.1	266	-	-	-	-
PCA5	251.3	283.2	229.1	261	194.8	226.7	244	275.9
PCA5+K	251.3	287.7	227.8	264.2	-	-	-	-
PCA6	250.9	287.3	218.3	**254.8**	193.9	230.3	244	280.4
PCA6+K	250.9	291.9	-	-	-	-	-	-
PCA7	239.1	280.1	215.7	256.6	191.6	232.6	243.7	284.6
PCA7+K	-	-	-	-	-	-	-	-
PCA8	237.8	283.3	211.2	256.8	191.1	236.6	241.9	287.4
PCA8+K	-	-	-	-	-	-	-	-

Models	SD/LD(V)		JIC/USC		JIC/USC(V)		VERN	
	Deviance	BIC	Deviance	BIC	Deviance	BIC	Deviance	BIC
Simple	268.9	**278**	274.4	283.5	273.1	282.3	282.9	292
K	266.9	280.6	261.4	275.1	252.3	266	251.3	265
Q8	256.1	301.6	206.2	251.7	182.6	228.1	198.9	244.4
Q8+K	256.1	306.1	205.6	255.7	181.9	232	191.2	241.2
nMDS8	245.7	291.2	215.8	261.3	199	244.6	204.6	250.2
nMDS+K	-	-	215	265.1	198.8	248.9	202.1	252.2
PCA1	268.1	281.8	243.4	257.1	269.6	283.3	267.7	281.4
PCA1+K	266.2	284.4	239.2	257.4	251	269.2	242.5	260.7

PCA2	262.6	280.8	236	254.2	233	251.3	228.2	246.4
PCA2+K	262.5	285.3	233.6	256.4	226.7	249.4	219.6	242.3
PCA3	260.3	283.1	231.6	254.4	187.3	210	216.6	239.3
PCA3+K	260.3	287.6	229.9	257.2	187	214.3	210.1	**237.4**
PCA4	258.1	285.4	231.2	258.5	184.7	212	216.4	243.8
PCA4+K	257.8	289.7	229.3	261.2	184.5	216.4	210.1	242
PCA5	254	285.8	223.2	255.1	176.8	**208.7**	214.8	246.6
PCA5+K	-	-	221.1	257.5	-	-	206.7	243.1
PCA6	253.9	290.3	223.2	259.7	176.7	213.1	208.2	244.6
PCA6+K	-	-	221.1	262	-	-	203	244
PCA7	252.3	293.3	196.8	**237.8**	176.5	217.4	208.1	249.1
PCA7+K	-	-	-	-	-	-	202.6	248.2
PCA8	252.1	297.7	196.1	241.7	176.4	222	205.9	251.4
PCA8+K	-	-	-	-	-	-	199.1	249.2

Notes: 1) - Denotes nonconvergence of the model. Q8 and nMDS8 represent ;2) Q8, nMDS8, and PCA8 represent the coordinates of the individual of 8 dimensions based on STRUTURE, nMDS, and PCA analysis, respectively, which are treated as fixed covariates in the regression models or linear mixed models.
