# Supplementary material for: Integrating Rare-Variant Testing, Function Prediction, and Gene Network in Composite Resequencing-Based Genome-Wide Association Studies (CR-GWAS)
Source: G3 (Bethesda). 2011 Aug 1;1(3):233–43. doi: 10.1534/g3.111.000364 (PMC3276137; doi:10.1534/g3.111.000364)
Supplement: Supporting Information [file supp_1.3.233_TableS6.pdf]

Table S6 Counts of SNPs in different MAF categories in the Arabidopsis dataset

MAF	Intronic	Synonymous	Benign	Possibly damaging	Probably damaging
0.000-0.025	2141	1517	1484	251	169
0.025-0.050	618	452	360	49	36
0.050-0.075	418	329	232	29	16
0.075-0.100	511	252	219	25	15
0.100-0.125	339	207	169	15	11
0.125-0.150	125	97	72	4	3
0.150-0.175	135	107	41	5	2
0.175-0.200	191	140	84	11	5
0.200-0.225	141	103	44	5	3
0.225-0.250	119	104	54	6	3
0.250-0.275	77	73	48	2	2
0.275-0.300	56	46	32	3	1
0.300-0.325	103	124	61	5	2
0.325-0.350	74	45	22	2	2
0.350-0.375	77	97	48	3	1
0.375-0.400	96	77	37	3	2
0.400-0.425	74	46	32	2	1
0.425-0.450	97	83	45	3	1
0.450-0.475	90	70	33	2	1
0.475-0.500	68	47	25	2	1
Total	5550	4016	3142	426	277

Note: In each MAF categories, the upper limit was included and the lower limit was excluded, 0.475-0.500 includes all SNPs with $0.475<MAF\leq 0.500$.
