# Supplementary material for: Integrating Rare-Variant Testing, Function Prediction, and Gene Network in Composite Resequencing-Based Genome-Wide Association Studies (CR-GWAS)
Source: G3 (Bethesda). 2011 Aug 1;1(3):233–43. doi: 10.1534/g3.111.000364 (PMC3276137; doi:10.1534/g3.111.000364)
Supplement: Supporting Information [file supp_1.3.233_TableS7.pdf]

Table S7 Values of r^2 among different bins of allele frequency along various chromosomes (standard deviations in parentheses). T1 to T6 correspond to SNP category (0, 0.05), [0.05, 0.1), [0.1, 0.2), [0.2, 0.3), [0.3, 0.4), (0.4, 0.5], respectively.

Chromosome		T1	T2	T3	T4	T5	T6
**1**	T1	0.1952(0.3875)	0.0312(0.1024)	0.0263(0.0554)	0.0199(0.0271)	0.0165(0.0183)	0.0182(0.0125)
	T2		0.6846(0.4404)	0.1272(0.2018)	0.1129(0.1071)	0.0804(0.0545)	0.0768(0.0305)
	T3			0.7325(0.3783)	0.3007(0.2854)	0.1431(0.1076)	0.1088(0.0648)
	T4				0.9028(0.2638)	0.4751(0.2616)	0.3152(0.1358)
	T5					0.8633(0.2352)	0.5846(0.2153)
	T6						0.8005(0.3408)
**2**	T1	0.1277(0.3174)	0.0195(0.0188)	0.0192(0.0474)	0.0187(0.0304)	0.0177(0.0762)	0.0174(0.0111)
	T2		0.4278(0.4683)	0.2288(0.2751)	0.0851(0.1038)	0.0697(0.0227)	0.0621(0.0424)
	T3			0.7747(0.3816)	0.2007(0.2247)	0.1843(0.1114)	0.1754(0.0576)
	T4				0.8222(0.3232)	0.3185(0.2351)	0.2765(0.0867)
	T5					0.9687(0.1186)	0.6340(0.2037)
	T6						0.9107(0.2017)
**3**	T1	0.1677(0.3656)	0.0273(0.0981)	0.0209(0.0458)	0.0207(0.0307)	0.0181(0.0118)	0.0169(0.0175)
	T2		0.6478(0.4553)	0.1785(0.2374)	0.1059(0.1196)	0.0822(0.0499)	0.0755(0.0261)
	T3			0.8916(0.2871)	0.2925(0.2897)	0.2120(0.1287)	0.2150(0.0523)
	T4				0.8509(0.2814)	0.2372(0.2461)	0.3656(0.1016)
	T5					0.8313(0.2992)	0.4719(0.1521)
	T6						0.8642(0.2307)
	T1	0.2313(0.4136)	0.0406(0.1128)	0.0317(0.0626)	0.0229(0.0306)	0.0181(0.0161)	0.0179(0.0108)
**4**	T2		0.6896(0.4369)	0.1154(0.2064)	0.1026(0.0993)	0.0789(0.0584)	0.0679(0.0263)
	T3			0.7199(0.4247)	0.2734(0.2697)	0.1569(0.1057)	0.1372(0.0511)
	T4				0.9615(0.1317)	0.2767(0.2497)	0.2916(0.0971)
	T5					0.9777(0.1063)	0.5833(0.1196)
	T6						0.9079(0.2399)
**5**	T1	0.2301(0.4135)	0.0231(0.0832)	0.0207(0.0472)	0.0173(0.0239)	0.0182(0.0181)	0.0159(0.0104)
	T2		0.4998(0.4831)	0.1114(0.2035)	0.0498(0.0675)	0.0636(0.0604)	0.0408(0.0342)
	T3			0.5925(0.4643)	0.1971(0.2579)	0.1158(0.1142)	0.0977(0.0764)
	T4				0.9159(0.2404)	0.2201(0.2288)	0.2531(0.1416)
	T5					0.8824(0.2445	0.3817(0.2646)
	T6						0.8217(0.3436)
