# Supplementary material for: Integrating Rare-Variant Testing, Function Prediction, and Gene Network in Composite Resequencing-Based Genome-Wide Association Studies (CR-GWAS)
Source: G3 (Bethesda). 2011 Aug 1;1(3):233–43. doi: 10.1534/g3.111.000364 (PMC3276137; doi:10.1534/g3.111.000364)
Supplement: Supporting Information [file supp_1.3.233_TableS8.pdf]

Table S8 Counts of fragments and SNPs across different chromosomes.

	Total		Common		Pooled-rare[a]		Combined[b]	
Chromosome	Fragments	SNPs	Fragments	SNPs	Fragments	SNPs	Fragments	SNPs[c]
1	329	5263	302	2282	308	2947	327	2589
2	200	3274	174	1544	185	1809	197	1726
3	239	3915	219	1835	223	2264	239	2050
4	230	4273	210	1946	222	2320	227	2168
5	277	4085	245	1864	256	2199	275	2120
Total	1275	20810	1153	9471	1194	11539	1265	10653

[a] denotes fragments whose number of rare variants are more than or equal to 3.

[b] includes any fragment either common variants or pooled-rare variant is present.

[c] indicates that pooled-rare variant is temporarily regarded as one SNP.
