# Supplementary material for: Integrating Rare-Variant Testing, Function Prediction, and Gene Network in Composite Resequencing-Based Genome-Wide Association Studies (CR-GWAS)
Source: G3 (Bethesda). 2011 Aug 1;1(3):233–43. doi: 10.1534/g3.111.000364 (PMC3276137; doi:10.1534/g3.111.000364)
Supplement: Supporting Information [file supp_1.3.233_TableS9.pdf]

**Table S9 Inflation factors by Genomic Control calculated for different statistical methods for 16 flowering time related traits**

| Traits | Single SNP | Pooled-rare variant | | | Multiple-SNP | CMP |
|---|---|---|---|---|---|---|
| | | Sum test | Weighted-sum | Function-aided | | |
| LD | 1.0981 | 1.1398 | 1.0672 | 1.0549 | 1.1552 | 1.1686 |
| LDV | 1.1453 | 1.0962 | 1.1650 | 1.0916 | 1.0298 | 1.1401 |
| SD | 1.2252 | 1.1213 | 1.0237 | 1.0744 | 1.1305 | 1.1643 |
| SDV | 1.1431 | 1.2252 | 1.0567 | 1.0384 | 1.2636 | 1.3046 |
| JIC0W | 1.2273 | 1.2434 | 1.1420 | 1.0184 | 1.3731 | 1.4343 |
| JIC2W | 1.1511 | 0.9674 | 0.9167 | 0.8341 | 1.2896 | 1.2525 |
| JIC4W | 1.1413 | 1.0507 | 0.9730 | 0.8792 | 1.1675 | 1.2342 |
| JIC8W | 1.0921 | 1.0648 | 1.0123 | 0.9072 | 1.3193 | 1.2126 |
| FLC | 1.1256 | 1.2288 | 1.1526 | 1.0683 | 1.1778 | 1.1449 |
| FRI | 1.0258 | 0.9206 | 0.9101 | 0.8201 | 0.8172 | 0.8093 |
| ±V(LD) | 1.0897 | 1.1162 | 1.0453 | 0.9676 | 1.0699 | 1.0991 |
| ±V(SD) | 1.0172 | 0.9931 | 0.9734 | 0.9094 | 0.9851 | 1.4423 |
| SD/LD(V) | 1.2421 | 1.1313 | 1.0871 | 1.0538 | 1.2224 | 1.2887 |
| JIC/USC | 1.2712 | 1.6392 | 1.5281 | 1.1756 | 1.0603 | 1.1605 |
| JIC/USC(V) | 1.0087 | 1.1616 | 1.0659 | 0.9883 | 1.1635 | 1.2219 |
| VERN | 1.2003 | 1.2024 | 1.0901 | 1.0032 | 1.2671 | 1.3019 |
