# Supplementary material for: Integrating Rare-Variant Testing, Function Prediction, and Gene Network in Composite Resequencing-Based Genome-Wide Association Studies (CR-GWAS)
Source: G3 (Bethesda). 2011 Aug 1;1(3):233–43. doi: 10.1534/g3.111.000364 (PMC3276137; doi:10.1534/g3.111.000364)
Supplement: Supporting Information [file supp_1.3.233_TableS11.pdf]

Table S11 Number of significant SNPs (MAF<0.5) at a Nominal of 10^{-5} level

Phenotype	Intergenic+perigenic	Intronic	Synonymous	Benign	Possibly damaging	Probably damaging	Total
LD	7	2	4	0	0	0	13
LDV	22	10	13	9	1	1	56
SD	5	0	0	0	0	0	5
SDV	31	20	18	12	1	3	85
JIC0W	0	0	4	0	0	0	4
JIC2W	4	4	9	8	1	0	26
JIC4W	25	13	20	S10	1	1	70
JIC8W	23	14	19	13	2	1	72
FLC	106	66	39	35	4	7	257
FRI	0	0	0	0	0	0	0
±V(LD)	7	0	2	1	0	0	10
±V(SD)	-1	0	0	0	0	0	-1
SD/LD(V)	54	27	32	19	2	3	137
JIC/USC	40	17	13	9	1	3	83
JIC/USC(V)	0	2	0	0	0	-1	1
VERN	5	0	5	0	0	0	10
Total	328	175	178	116	13	18	828
