# Supplementary material for: Integrating Rare-Variant Testing, Function Prediction, and Gene Network in Composite Resequencing-Based Genome-Wide Association Studies (CR-GWAS)
Source: G3 (Bethesda). 2011 Aug 1;1(3):233–43. doi: 10.1534/g3.111.000364 (PMC3276137; doi:10.1534/g3.111.000364)
Supplement: Supporting Information [file supp_1.3.233_TableS13.pdf]

Table S13 Top 30 significant associations between multiple-common variants and flowering-time traits

Chromosome	Position	Gene ID	Functional prediction (PolyPhen)		Trait	LR value	-log10(p)
			Position	Function			
5	26798104	AT5G67160			SDV	48.56	11.49
3	483006	AT3G02360			SDV	35.16	8.51
4	6291471				LDV	24.89	4.91
1	2398546	AT1G07740			SDV	18.4	4.77
1	7341625				LDV	26.05	5.1
1	26569524				SDV	21.79	4.91
1	9441536	AT1G27180			SDV	19.86	5.09
3	4056760	AT3G12770			SDV	18.29	4.72
2	19301478	AT2G46980			SDV	24.35	6.09
1	3872234	AT1G11510	3871281	Probably damaging	SDV	18.33	4.73
5	7442036	AT5G22450			±V(SD)	11.76	3.21
5	26798104	AT5G67160			±V(SD)	13.62	3.65
5	21500115	AT5G53020			JIC/USC(V)	15.13	3.99
2	1737439	AT2G04940			SDV	17.64	4.57
1	4991932	AT1G14580			SDV	15.96	4.18
3	777470	AT3G03300			SDV	14.48	3.85
3	8753334	AT3G24210			±V(SD)	13.22	3.55
1	394740	AT1G02110			SDV	15.26	4.02
1	20580206	AT1G55170			SDV	18.58	4.78
5	6416241	AT5G19130			JIC2W	20.57	5.24
5	6416241	AT5G19130			SD	19.26	4.94
4	6780974	AT4G11130	6781455	Possibly damaging	SDV	15.77	4.14
5	6221771	AT5G18660			VERN	15.88	4.17
5	19698494	AT5G48590			FRI	11.39	3.13
1	8384902	AT1G23710			SDV	18.75	4.82
5	10102071	AT5G28090			SDV	15.43	4.06
3	6400099	AT3G18600			FLC	11.89	3.24
3	22304742	AT3G60340			SDV	19.43	4.98
4	9330327	AT4G16560			SDV	15.46	4.07
5	6416241	AT5G19130			SDV	19.55	5.01

Notes: 1) All the significant *a priori* candidate genes are excluded from this list; 2) *P* values are computed on the assumption that LR approximately follows Chi-square distribution with 1 degree of freedom.
