# Supplementary material for: Integrating Rare-Variant Testing, Function Prediction, and Gene Network in Composite Resequencing-Based Genome-Wide Association Studies (CR-GWAS)
Source: G3 (Bethesda). 2011 Aug 1;1(3):233–43. doi: 10.1534/g3.111.000364 (PMC3276137; doi:10.1534/g3.111.000364)
Supplement: Supporting Information [file supp_1.3.233_TableS14.pdf]

Table 14 Top 30 significant associations between pooled-rare variant and flowering-time traits

Chromosome	Genes ID	No. SNP	Functional prediction (PolyPhen)		Trait	LR	-log10(p)
			Position	Function			
2	AT2G11000	7			JIC/USC(V)	23.30	5.86
5	AT5G38840	17			JIC4W	37.93	9.13
3	AT3G18860	6			SD	18.73	4.82
2	AT2G11000	7			JIC4W	27.71	6.85
5	AT5G67160	3			SDV	28.99	7.14
5	AT5G38840	17			SDV	23.37	5.87
4	AT4G00730	4			SD	20.89	5.31
4	AT4G21250	13			±V(SD)	14.41	3.83
5	AT5G38840	17			JIC/USC(V)	18.45	4.76
5	AT5G38840	17			JIC8W	30.54	7.48
3	AT3G18860	6			JIC8W	19.52	5
5	AT5G38840	17			JIC0W	20.23	5.16
5	AT5G39080	26	15642453,15642447 15642405	Possibly damaging	JIC/USC	15.99	3.51
5	AT5G42180	10			SDV	20.49	5.22
5	AT5G35950	14			JIC/USC	15.23	4.02
3	AT3G15120	5			SD	16.90	4.41
5	AT5G38840	17			JIC2W	23.69	5.95
5	AT5G47910	7			JIC8W	21.76	5.51
4	AT4G07390	24			JIC/USC	13.57	3.64
5	AT5G52500	6	21301806,21301857 21302035	Possibly damaging	JIC/USC	13.82	3.7
5	AT5G38840	17			SD	17.47	4.54
4	AT4G13360	62	7777202,7777216 7776218	Probably damaging	JIC/USC	15.58	4.1
2	AT2G11000	7			JIC2W	14.64	3.89
5	AT5G47910	7			JIC4W	18.81	4.84
5	AT5G43420	31	17452135,17452150 17452154,17452235 17452262	Possibly damaging	JIC2W	14.65	3.89
5	AT5G48850	4			JIC0W	14.86	3.94
4	AT4G13360	62			±V(SD)	12.51	3.39
5	AT5G47910	7			JIC2W	17.93	4.64
3	AT3G15120	5			JIC0W	14.38	3.83
3	AT3G15120	5			JIC2W	13.81	3.69

Notes: 1) All the significant *a priori* candidate genes are excluded from this list; 2) P values are computed on the assumption that LR (from the weighted sum test) approximately follows Chi-square distribution with 1 degree of freedom.
