# Supplementary material for: Integrating Rare-Variant Testing, Function Prediction, and Gene Network in Composite Resequencing-Based Genome-Wide Association Studies (CR-GWAS)
Source: G3 (Bethesda). 2011 Aug 1;1(3):233–43. doi: 10.1534/g3.111.000364 (PMC3276137; doi:10.1534/g3.111.000364)
Supplement: Supporting Information [file supp_1.3.233_TableS15.pdf]

Table S15 Rare sequence variations in FLM, SPL5, and FY. As for nonsynonymous variants, the effect of each amino acid substitution on protein function was predicted with the use of PolyPhen.

Genes	Position	Minor	Major	MAF	Amino acid change	Predicted effect
FLM	28955675	C	A	0.0104		5`UTR
(AT1G77080)	28955719	G	A	0.0104	K>R	Benign
	28955851	T	C	0.0104	S>F	Benign
	28955854	A	C	0.0208	S>Y	Possibly damaging
	28956003	A	C	0.0104		Intron
	28958360	G	A	0.0104	E>G	Benign
	28958426	A	C	0.0417		Intron
	28958437	A	G	0.0104		Intron
	28958510	T	G	0.0104		Intron
	28958524	T	A	0.0104		Intron
	28958719	C	G	0.0417		Intron
	28958908	C	A	0.0208		Intron
	28958910	T	C	0.0104		Intron
	28958968	A	G	0.0104	E>K	Benign
SPL5	5140959	C	T	0.0104	R>G	Benign
(AT3G15270)	5141109	T	C	0.0104	V>I	Benign
	5141147	T	C	0.0104	G>E	Possibly damaging
	5141191	G	A	0.0104		Synonymous
	5141207	A	G	0.0208	T>I	Benign
	5141246	C	T	0.0417	Q>R	Benign
	5141247	C	G	0.0104	Q>E	Benign
FY	4327374	C	T	0.0312	V>M	Benign
(AT5G13480)	4327530	T	G	0.0104	M>L	Benign
	4327531	A	T	0.0104		Synonymous
	4327847	A	G	0.0104		Intron
	4329900	A	G	0.0104		Intron
	4330057	T	C	0.0208		Intron
	4330115	A	T	0.0208		Intron
	4330383	A	T	0.0104		Synonymous
BAS1	11382307	A	G	0.0421		Intron
(AT2G26710)	11382319	G	T	0.0315		Intron
	11382342	T	A	0.0421		Intron
