# Supplementary material for: Integrating Rare-Variant Testing, Function Prediction, and Gene Network in Composite Resequencing-Based Genome-Wide Association Studies (CR-GWAS)
Source: G3 (Bethesda). 2011 Aug 1;1(3):233–43. doi: 10.1534/g3.111.000364 (PMC3276137; doi:10.1534/g3.111.000364)
Supplement: Supporting Information [file supp_1.3.233_TableS16.pdf]

Table S16 Total 161 seed genes are connected one another.

AT1G01040	AT1G04400	AT1G06040	AT1G09530	AT1G09570	AT1G09700	AT1G14400	AT1G14920
AT1G18450	AT1G22770	AT1G24260	AT1G25540	AT1G26310	AT1G30960	AT1G30970	AT1G43700
AT1G43850	AT1G44446	AT1G61040	AT1G62750	AT1G62830	AT1G65480	AT1G66350	AT1G68050
AT1G69120	AT1G71692	AT1G72050	AT1G72830	AT1G77080	AT1G78300	AT1G78440	AT1G79280
AT1G79460	AT1G79730	AT2G01570	AT2G02560	AT2G02950	AT2G03710	AT2G06210	AT2G18790
AT2G18915	AT2G19520	AT2G20180	AT2G22540	AT2G22630	AT2G23380	AT2G24790	AT2G25930
AT2G26710	AT2G28550	AT2G32950	AT2G40080	AT2G42830	AT2G43010	AT2G44680	AT2G45650
AT2G45660	AT2G46790	AT2G46830	AT3G02310	AT3G02885	AT3G05120	AT3G06910	AT3G06930
AT3G10390	AT3G11540	AT3G12810	AT3G13682	AT3G15270	AT3G15354	AT3G19040	AT3G19980
AT3G20550	AT3G20740	AT3G24440	AT3G26640	AT3G33520	AT3G46640	AT3G47500	AT3G48430
AT3G54560	AT3G54990	AT3G57230	AT3G57300	AT3G57390	AT3G58780	AT3G59060	AT3G60250
AT3G61120	AT3G62090	AT3G63010	AT4G00650	AT4G00690	AT4G02020	AT4G03400	AT4G04890
AT4G08920	AT4G11110	AT4G11880	AT4G15880	AT4G16250	AT4G16780	AT4G16845	AT4G17640
AT4G18130	AT4G20370	AT4G22950	AT4G24540	AT4G25530	AT4G29130	AT4G31120	AT4G31500
AT4G32551	AT4G32980	AT4G35900	AT4G37940	AT5G02840	AT5G04240	AT5G05690	AT5G10140
AT5G11260	AT5G13480	AT5G13790	AT5G14920	AT5G15800	AT5G15840	AT5G15850	AT5G15960
AT5G15970	AT5G17690	AT5G20730	AT5G24470	AT5G25900	AT5G27230	AT5G27320	AT5G35840
AT5G37020	AT5G37055	AT5G39660	AT5G41360	AT5G41790	AT5G46210	AT5G47010	AT5G47080
AT5G49020	AT5G51230	AT5G51820	AT5G57360	AT5G57380	AT5G58230	AT5G59710	AT5G60910
AT5G61150	AT5G61380	AT5G62430	AT5G64813	AT5G65050	AT5G65060	AT5G65070	AT5G65080
AT5G67100							
