# Supplementary material for: Integrating Rare-Variant Testing, Function Prediction, and Gene Network in Composite Resequencing-Based Genome-Wide Association Studies (CR-GWAS)
Source: G3 (Bethesda). 2011 Aug 1;1(3):233–43. doi: 10.1534/g3.111.000364 (PMC3276137; doi:10.1534/g3.111.000364)
Supplement: Supporting Information [file supp_1.3.233_TableS17.pdf]

Table S17 Total 99 seed genes are disconnected one another.

AT1G01060	AT1G04440	AT1G13260	AT1G15550	AT1G19850	AT1G22690	AT1G26260	AT1G30040
AT1G30950	AT1G31814	AT1G44090	AT1G48270	AT1G50960	AT1G52800	AT1G53090	AT1G53160
AT1G55080	AT1G57820	AT1G59940	AT1G60980	AT1G65380	AT1G69935	AT1G70170	AT1G74670
AT1G76710	AT1G77300	AT1G80330	AT1G80340	AT1G80680	AT2G04030	AT2G21070	AT2G27550
AT2G28290	AT2G34555	AT2G39250	AT2G39810	AT2G43410	AT2G46340	AT2G47310	AT2G47700
AT3G01460	AT3G02380	AT3G03090	AT3G04610	AT3G05040	AT3G07650	AT3G11440	AT3G18990
AT3G22380	AT3G30180	AT3G54720	AT3G58070	AT4G00450	AT4G02560	AT4G02780	AT4G12480
AT4G14110	AT4G14690	AT4G15090	AT4G15180	AT4G16280	AT4G21200	AT4G21690	AT4G22140
AT4G23340	AT4G24620	AT4G25420	AT4G27430	AT4G29830	AT4G30200	AT4G32040	AT4G36920
AT4G37580	AT4G39400	AT5G02810	AT5G03790	AT5G03840	AT5G06100	AT5G07200	AT5G08330
AT5G11530	AT5G12840	AT5G15230	AT5G19550	AT5G23150	AT5G25810	AT5G28450	AT5G37770
AT5G38150	AT5G46910	AT5G51310	AT5G51810	AT5G59570	AT5G59845	AT5G60100	AT5G60120
AT5G62040	AT5G62640	AT5G65540					
