# Supplementary material for: Integrating Rare-Variant Testing, Function Prediction, and Gene Network in Composite Resequencing-Based Genome-Wide Association Studies (CR-GWAS)
Source: G3 (Bethesda). 2011 Aug 1;1(3):233–43. doi: 10.1534/g3.111.000364 (PMC3276137; doi:10.1534/g3.111.000364)
Supplement: Supporting Information [file supp_1.3.233_TableS18.pdf]

Table S18 Total 33 valid Arabidopsis seed genes but NOT found in AraNet.

AT1G10588	AT1G63030	AT2G33810	AT3G04510	AT3G26120	AT4G16810	AT5G02200	AT5G28490
AT1G121910	AT1G74660	AT2G33835	AT3G05690	AT3G26790	AT4G27060	AT5G10625	AT5G48150
AT1G12610	AT2G14900	AT2G37678	AT3G10185	AT4G01060	AT4G31380	AT5G16320	AT5G54510
AT1G49480	AT2G30810	AT2G39540	AT3G21320	AT4G09610	AT4G33280	AT5G24860	AT5G59560
AT5G61850							
