# Supplementary material for: Integrating Rare-Variant Testing, Function Prediction, and Gene Network in Composite Resequencing-Based Genome-Wide Association Studies (CR-GWAS)
Source: G3 (Bethesda). 2011 Aug 1;1(3):233–43. doi: 10.1534/g3.111.000364 (PMC3276137; doi:10.1534/g3.111.000364)
Supplement: Supporting Information [file supp_1.3.233_TableS20.pdf]

Table 20 Associated genes to 150 valid seed gene(s) by AraNet that are also among Top 30 statistically significant.

Rank	Gene ID	Paralogs	Symbol	Score	Evidence	Linked seeds	Linked genes	GO_P	GO_C	GO_F
148	AT3G03300	No paralog	DCL2	6.3	HS-DC:0.42 AT-GN:0.31 AT-DC:0.27	4/150	HYL1,AT1G30960 XPB2,AT5G47010	RNA interference, production of ta-siRNAs; miRNA-mediated gene silencing, production of miRNAs;	intracellular	ribonuclease III activity; ATP-dependent helicase activity;
586	AT4G00730	AT3G61150	ANL2	4.73	HS-LC:0.76 HS-DC:0.17 CE-CC:0.08	12/150	AT1G72050,AGL17,PIF4,AGL16,AGL18,ATHB-2,VRN2,AGL24,AGL15, EMF2, AGL31, MAF4	anthocyanin accumulation in tissues in response to UV light; root development;	nucleus	transcription factor activity; transcription regulator activity;
1525	AT3G18660	No paralog	NA	3.2	CE-CX:0.85 HS-LC:0.15	6/150	AT3G05120, AT3G63010, ATHXK1, AT5G27320, PGM MSI1	biosynthetic process;	chloroplast	transferase activity, transferring glycosyl groups;
3361	AT3G02360	No paralog	NA	1.63	SC-CC:1.00	2/150	AT4G31120, AT5G46210	pentose-phosphate shunt;	endomembrane system	phosphoglucoNAte dehydrogeNAse activity;
4086	AT2G11000	No paralog	ATMAK10	1.18	CE-CX:1.00	1/150	ESD4	NA	NA	acetyltransferase activity;
4713	AT3G18600	No paralog	NA	0.95	HS-CX:1.00	1/150	EZA1	NA	NA	ATP-dependent helicase activity;
5143	AT3G15120	No paralog	NA	0.89	AT-CX:1.00	1/150	AT3G57300	NA	NA	ATPase activity;
5386	AT5G48850	No paralog	NA	0.86	AT-CX:1.00	1/150	AT5G27230	NA	NA	NA

Notes*: GO_P: GO biological process, GO_C: GO cellular component, GO_F: GO molecular function*
