# Supplementary material for: Integrating Rare-Variant Testing, Function Prediction, and Gene Network in Composite Resequencing-Based Genome-Wide Association Studies (CR-GWAS)
Source: G3 (Bethesda). 2011 Aug 1;1(3):233–43. doi: 10.1534/g3.111.000364 (PMC3276137; doi:10.1534/g3.111.000364)
Supplement: Supporting Information [file supp_1.3.233_TableS21.pdf]

Table S21 6 Seed genes connected to one another in AraNet (ranked by total connectivity).

Rank	Gene ID	Symbol	Score	Evidence	Linked-seeds	Linked-genes	GO_P	GO_C	GO_F
1	AT1G30960	NA	7.23	AT-GN:1.00	3/6	HYL1<br>DCL1<br>DCL2	NA	Intracellular;;	GTP binding
2	AT3G03300	DCL1	6.84	HS-DC:0.42<br>AT-GN:0.31<br>AT-DC:0.27<br>AT-LC:0.19	4/6	HYL1<br>AT1G30960<br>XPB2<br>AT5G47010	RNA interference;<br>Production of ta-siRNAs;<br>miRNA-mediated gene<br>production of miRNAs. RNA	Intracellular	Ribonuclease III activity,<br>aTP-dependent helicase
3	AT1G09700	HYL1	6.38	AT-GN:0.54<br>AT-DC:0.46	3/6	AT1G30960<br>DCL2<br>DCL1	Response to auxin stimulus;<br>response to cytokinin stimulus;<br>response to abscisic acid<br>miRNA-mediated gene<br>production of miRNAs; mRNA<br>miRNA-mediated gene	Nucleus	Double-stranded RNA<br>protein binding
4	AT5G47010	NA	6.32	HS-DC:0.60<br>AT-DC:0.40	2/6	DCL2<br>XPB2	Response to sucrose stimulus;<br>sugar mediated signaling;	NA	RNA helicase activity
5	AT3G03300	DCL2	6.30	HS-DC:0.42<br>AT-GN:0.31<br>AT-DC:0.27	4/6	HYL1<br>AT1G30960<br>XPB2<br>AT5G47010	RNA interference ,<br>Production of ta-siRNAs;<br>miRNA-mediated gene<br>production of miRNAs. RNA	intracellular	Ribonuclease III activity,<br>ATP-dependent helicase
6	AT5G41360	XPB2	6.21	HS-DC:0.69<br>AT-DC:0.31	3/6	DCL1<br>DCL2<br>AT5G47010	response to UV-B;	nucleus	ATP-dependent helicase

Notes*: GO_P: GO biological process, GO_C: GO cellular component, GO_F: GO molecular function*
