# Supplementary material for: Integrating Rare-Variant Testing, Function Prediction, and Gene Network in Composite Resequencing-Based Genome-Wide Association Studies (CR-GWAS)
Source: G3 (Bethesda). 2011 Aug 1;1(3):233–43. doi: 10.1534/g3.111.000364 (PMC3276137; doi:10.1534/g3.111.000364)
Supplement: Supporting Information [file supp_1.3.233_TableS22.pdf]

Table S22 Evidence codes for 24 types of data sets incorporated in AraNet.

Evidence code	Data set description	Evidence code	Data set description
AT-CX	Co-expression among Arabidopsis genes	HS-DC	Co-occurrence of domains among human proteins
AT-DC	Co-occurrence of domains among Arabidopsis proteins	HS-LC	Literature curated human protein physical interactions
AT-GN	Gene neighborhoods of bacterial and archaeal orthologs of	HS-MS	human protein complexes from affinity purification/mass
AT-LC	Literature curated Arabidopsis protein physical interactions	HS-YH	High-throughput yeast 2-hybrid assays among human genes
AT-PG	Co-inheritance of bacterial and archaeal orthologs of Arabidopsis	SC-CC	Co-citation of yeast genes
CE-CC	Co-citation of worm gene	SC-CX	Co-expression among yeast genes
CE-CX	Co-expression among worm genes	SC-DC	Co-occurrence of domains among yeast proteins
CE-GT	Worm genetic interactions	SC-GT	Yeast genetic interactions
CE-LC	Literature curated worm protein physical interactions	SC-LC	Literature curated yeast protein physical interactions
CE-YH	High-throughput yeast 2-hybrid assays among worm genes	SC-MS	Yeast protein complexes from affinity purification/mass
DM-PI	Fly protein physical interactions	SC-TS	Yeast protein interactions inferred from tertiary structures of
HS-CX	Co-expression among human genes	SC-YH	High-throughput yeast 2-hybrid assays among yeast
